# MAPK Signaling‐Mediated RFNG Phosphorylation and Nuclear Translocation Restrain Oxaliplatin‐Induced Apoptosis and Ferroptosis

**DOI:** 10.1002/advs.202402795

**Published:** 2024-08-09

**Authors:** Yuqin Di, Xiang Zhang, Xiangqiong Wen, Jiale Qin, Lvlan Ye, Youpeng Wang, Mei Song, Ziyang Wang, Weiling He

**Affiliations:** ^1^ Molecular Diagnosis and Gene Testing Center The First Affiliated Hospital Sun Yat‐sen University Guangzhou Guangdong 510080 China; ^2^ Department of Gastrointestinal Surgery The First Affiliated Hospital Sun Yat‐sen University Guangzhou Guangdong 510080 China; ^3^ Department of Biochemistry Zhongshan School of Medicine Sun Yat‐sen University Guangzhou Guangdong 510080 China; ^4^ Center of Hepato‐Pancreato‐Biliary Surgery The First Affiliated Hospital Sun Yat‐sen University Guangzhou Guangdong 510080 China; ^5^ Institute of Precision Medicine The First Affiliated Hospital Sun Yat‐Sen University Guangzhou Guangdong 510080 China; ^6^ Center for Translational Medicine The First Affiliated Hospital Sun Yat‐sen University Guangzhou Guangdong 510080 China; ^7^ Department of Gastrointestinal Surgery Xiang'an Hospital of Xiamen University School of Medicine Xiamen University Xiamen Fujian 361000 China

**Keywords:** apoptosis and ferroptosis, MAPK signaling, nuclear RFNG, oxaliplatin chemosensitivity, p53 activity

## Abstract

Chemotherapy resistance remains a major challenge in the treatment of colorectal cancer (CRC). Therefore, it is crucial to develop novel strategies to sensitize cancer cells to chemotherapy. Here, the fringe family is screened to determine their contribution to chemotherapy resistance in CRC. It is found that RFNG depletion significantly sensitizes cancer cells to oxaliplatin treatment. Mechanistically, chemotherapy‐activated MAPK signaling induces ERK to phosphorylate RFNG Ser255 residue. Phosphorylated RFNG S255 (pS255) interacts with the nuclear importin proteins KPNA1/importin‐α1 and KPNB1/importin‐β1, leading to its translocation into the nucleus where it targets p53 and inhibits its phosphorylation by competitively inhibiting the binding of CHK2 to p53. Consequently, the expression of CDKN1A is decreased and that of SLC7A11 is increased, leading to the inhibition of apoptosis and ferroptosis. In contrast, phosphor‐deficient RFNG S225A mutant showed increased apoptosis and ferroptosis, and exhibited a notable response to oxaliplatin chemotherapy both in vitro and in vivo. It is further revealed that patients with low RFNG pS255 exhibited significant sensitivity to oxaliplatin in a patient‐derived xenograft (PDX) model. These findings highlight the crosstalk between the MAPK and p53 signaling pathways through RFNG, which mediates oxaliplatin resistance in CRC. Additionally, this study provides guidance for oxaliplatin treatment of CRC patients.

## Graphical abstract

This study shows that chemotherapy‐activated MAPK signaling induces ERK to phosphorylate RFNG Ser255 residue. RFNG pS255 enhances chemoresistance to oxaliplatin by translocating into the nucleus, where it targets p53 to inhibit apoptosis and ferroptosis. Patients with low RFNG pS255 levels exhibit significant sensitivity to chemotherapy in a patient‐derived‐xenograft (PDX) model, offering valuable insights for the treatment of oxaliplatin.

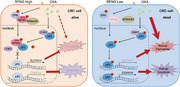

## 1 Introduction

Colorectal cancer (CRC) is the third most common cancer worldwide and the second leading cause of cancer‐related deaths.^[^ ^1^ ^]^ Chemotherapy plays a crucial role in the treatment of CRC, and preoperative neoadjuvant chemotherapy, surgery, and postoperative adjuvant chemotherapy are important treatment options for patients with advanced CRC.^[^ ^2^ ^]^ Oxaliplatin, a third‐generation platinum chemotherapy drug, is the first‐line chemotherapeutic drug for CRC treatment.^[^ ^3^ ^]^ Although oxaliplatin‐containing chemotherapy regimens have shown improved response rates in patients with CRC, the development of chemotherapy resistance remains a significant challenge.^[^ ^4^ ^]^ Therefore, it is crucial to identify targets that enhance cancer cell sensitivity to chemotherapy and explore strategies to reduce side effects and drug resistance.

In humans, the *p53* tumor suppressor gene (also known as *TP53*) is often referred to as the “genome guardian” or “cell guardian”.^[^ ^5^ ^]^ Under exposure to various stressors, p53 can be activated to perform different functions, including its well‐known roles in inducing apoptosis, maintaining genomic stability, regulating metabolism, and regulating autophagy. Additionally, emerging research suggests that p53 plays a role in the regulation of ferroptosis, a form of cell death.^[^ ^6^ ^]^ During chemotherapy, the activation of p53 in tumor cells regulates important processes such as cell apoptosis and cell cycle regulation. One way that p53 promotes apoptosis by inducing CDKN1A (also known as p21) expression. CDKN1A inhibits cell cycle progression by preventing cells from entering the S and G2/M phases. CDKN1A also interacts with proteins involved in cell apoptosis, such as the Bcl‐2 family inhibitor, Bax, which promotes tumor cell apoptosis.^[^ ^7^ ^]^ Furthermore, studies have shown that p53 transcriptionally inhibits SLC7A11, a key cysteine/glutamate reverse transporter protein, leading to inhibition of cysteine uptake and increased sensitivity of cells to ferroptosis.^[^ ^8^ ^]^ Although p53 is highly mutated in different tumor types, its functions are often repressed in many tumors with wild‐type (WT) p53, making tumor cells resistant to chemotherapy. Therefore, studying the mechanisms of WT p53 inhibition and reactivation of compromised p53 could benefit many cancer patients.

Fringes are glycosyltransferases responsible for transferring N‐acetylglucosamine to the O‐linked fucose of Notch receptors.^[^ ^3^ ^]^ In humans, there are three known fringe members: lunatic fringe (LFNG), manic fringe (MFNG), and radical fringe (RFNG). These fringes have been identified as crucial regulators of the Notch signaling pathway, which plays a significant role in various cancers.^[^ ^9^, ^10^ ^]^ Recent research has elucidated the impact of fringe proteins in cancer, revealing that they can have dual functions, acting as either oncogenes or tumor suppressors, depending on the specific cancer type. For example, LFNG has been found to function as a tumor suppressor,^[^ ^11^ ^]^ while MFNG acts as an oncogene in breast cancer.^[^ ^12^ ^]^ In addition, LFNG has been implicated in promoting CRC progression through its involvement in the activation of Notch signaling.^[^ ^13^ ^]^ However, most studies have focused on the enzymatic activity of fringe members in modifying Notch proteins, and it remains unclear whether fringe members play a role in CRC chemoresistance through non‐enzymatic mechanisms.

In this study, we identified RFNG as a potential critical chemoresistance factor in CRC. We showed that, under oxaliplatin treatment, CRC cells undergo RFNG phosphorylation at the Ser255 residue through the MAPK kinase ERK. This phosphorylation event leads to the translocation of RFNG into the nucleus via its binding to nuclear importin proteins. In the nucleus, RFNG interacts with p53 and inhibits its activity, resulting in downregulation of CDKN1A expression and upregulation of SLC7A11 expression. This leads to inhibition of apoptosis and ferroptosis. Our study highlights the potential of RFNG phosphorylation as a biomarker for predicting the efficacy of oxaliplatin chemotherapy and suggests that targeting RFNG could be a promising strategy for overcoming chemoresistance in CRC.

## 2 Results

### 2.1 RFNG Promotes Oxaliplatin Chemoresistance and Serves as a Prognostic Biomarker in CRC

**Figure 1:**
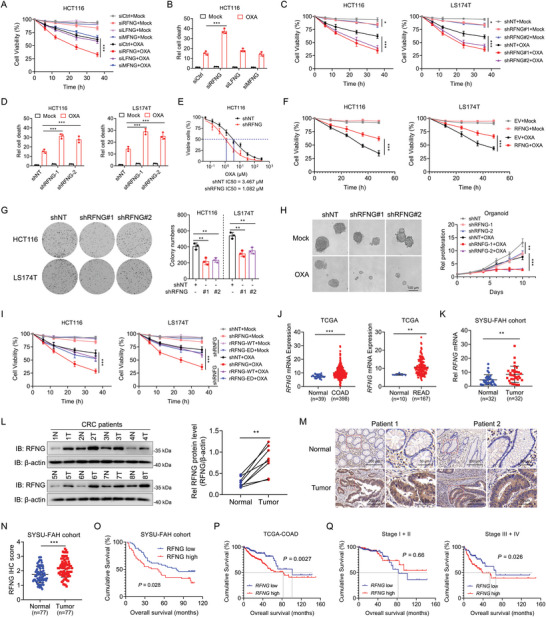
RFNG promotes oxaliplatin chemoresistance and serves as a prognostic biomarker in CRC. A) Cell viability of HCT116 cells transfected with siCtril, siRFNG, siLFNG, or siMFNG and treated with or without 20 µm oxaliplatin (OXA) for the indicated times. B) HCT116 cells transfected with siCtril, siRFNG, siLFNG or siMFNG were treated with or without 20 µm OXA for 24 h and collected for cell death measurement. C‐E) HCT116 and LS174T cells stably expressing shNT, shRFNG‐1, or shRFNG‐2 were treated with or without 20 µm OXA for the indicated times, and cell viability was assessed (C). Cell death was measured after treatment for 24 h (D), and the IC50 of HCT116 cells was assessed after treatment for 48 h (E). F) HCT116 and LS174T cells stably expressing empty vector (EV) or RFNG were treated with or without 20 µM OXA for the indicated times, and cell viability was assessed. G) Colony formation assays were conducted to assess the proliferation of HCT116 and LS174T cells stably expressing shNT, shRFNG‐1, or shRFNG‐2. H) Representative images (left) and growth curves (right) of shNT, shRFNG‐1, or shRFNG‐2 CRC organoids treated with or without 10 µm OXA for the indicated times. I) HCT116 and LS174T cells stably expressing shNT, shRFNG, or shRFNG rescued with rRFNG‐WT or rRFNG‐ED were treated with or without 20 µm OXA for the indicated times, and cell viability was assessed. J) The mRNA expression of *RFNG* in normal and tumor samples from the TCGA COAD and READ databases. K) QPCR analysis of *RFNG* mRNA expression in 32 CRC tumor tissues and paired normal tissues. L) Immunoblotting analysis of RFNG protein expression in paired CRC tissues. M,N) Representative images (M) and IHC scores (N) of IHC staining of RFNG in CRC tissues and paired normal tissues. O,P) Kaplan‒Meier analysis of overall survival according to RFNG expression in CRC patient samples from the SYSU‐FAH cohort (O) and the TCGA COAD database (P). Q) Kaplan‒Meier analysis of overall survival according to RFNG expression in stage I+II (left) or stage III+IV (right) CRC patient samples from the TCGA COAD database. **P < 0.01, ***P < 0.001 (two‐way ANOVA (A, C, F, H, I), one‐way ANOVA (B, D, G), two‐tailed t test (J, K, L, N), or log‐rank test (O, P, Q)).

To investigate the relationship between fringe family members and chemosensitivity in CRC, we first knocked down fringe family genes (RFNG, LFNG, and MFNG) in HCT116 cells (Figure S1A, Supporting Information). Subsequently, we treated these cells with oxaliplatin and observed that RFNG depletion significantly increased the sensitivity of CRC cells to oxaliplatin, whereas depletion of other fringe family members had little impact on chemotherapy resistance (**Figure** **1**A). These findings were supported by the finding that only cells with RFNG knockdown showed increased cell death when exposed to oxaliplatin (Figure 1B). To further validate the role of RFNG in chemoresistance, we used two shRNAs targeting RFNG to knock down RFNG in HCT116 and LS174T cells (Figure S1B, Supporting Information). Additionally, we confirmed that RFNG depletion did not affect the expression of other fringe family genes (Figure S1C, Supporting Information). As expected, RFNG depletion enhanced the sensitivity of both HCT116 and LS174T cells to oxaliplatin (Figure 1C). RFNG‐deficient cells also exhibited increased cell death and lower half maximal inhibitory concentration (IC50) values, indicating reduced chemoresistance in response to oxaliplatin (Figure 1D,E; Figure S1D, Supporting Information).

To further investigate the role of RFNG in CRC chemoresistance, we conducted gain‐of‐function assays by overexpressing RFNG in HCT116 and LS174T cells (Figure S1E, Supporting Information). RFNG overexpression significantly promoted resistance to oxaliplatin (Figure 1F). In addition, RFNG overexpression reduced cell death and increased IC50 values (Figure S1F,G, Supporting Information). We also observed that RFNG knockdown inhibited CRC cell proliferation (Figure 1G; Figure S1H, Supporting Information), and RFNG overexpression promoted CRC cell proliferation (Figure S1I,J, Supporting Information). We then detected RFNG expression in several CRC cell lines, namely HCT116, RKO, LS174T, and LoVo cells, demonstrating the lowest expression of RFNG in RKO cells and the highest expression of RFNG in LoVo cells (Figure S1K, Supporting Information). We also overexpressed RFNG in RKO and LoVo cells (Figure S1L, Supporting Information). The results revealed that oxaliplatin‐induced cell death was more effective in RKO cells compared to LoVo cells, indicating that cell lines with low RFNG expression are more sensitive to oxaliplatin stimulation. Additionally, we found that RFNG overexpression reduced the sensitivity of CRC cells to oxaliplatin stimulation (Figure S1G,M, Supporting Information). These findings provide further evidence supporting the role of RFNG in mediating the sensitivity of CRC cells to oxaliplatin. Furthermore, the combination of RFNG depletion and oxaliplatin treatment significantly suppressed the growth of the CRC patient‐derived organoids (Figure 1H). To explore whether RFNG metabolic activity is essential for its function in CRC chemoresistance, we restored RFNG expression using a synonymously mutated shRNA‐resistant RFNG wild‐type (rRFNG WT) and an enzymatically dead (ED) rRFNG variant in RFNG‐depleted HCT116 and LS174T cells (Figure S1N, Supporting Information). The results showed that both rRFNG WT and ED fully restored the RFNG depletion‐enhanced sensitivity of CRC cells to oxaliplatin, excluding the possibility of off‐target effects of RFNG shRNAs, which indicated that RFNG metabolic activity is not essential for its function in CRC chemoresistance (Figure 1I; Figure S1O, Supporting Information). Overall, these findings provide further evidence of the crucial role of RFNG in promoting oxaliplatin chemoresistance in CRC.

We investigated the clinical implications of RFNG in patients with CRC. We analyzed the information on *RFNG* expression in colon adenocarcinoma (COAD) and rectal adenocarcinoma (READ) from The Cancer Genome Atlas (TCGA) database and found that RFNG mRNA levels were much higher in tumor tissues than in normal tissues (Figure 1J). Higher mRNA levels of *RFNG* in CRC were also confirmed in the Gene Expression Omnibus (GEO) datasets GSE9348,^[^ ^14^ ^]^ GSE39582^[^ ^15^ ^]^ and GSE44076^[^ ^16^ ^]^ (Figure S1P, Supporting Information). We further performed quantitative PCR (qPCR), immunoblotting, and immunohistochemistry (IHC) analyses and observed a notable increase in RFNG expression in tumor tissues compared to paired normal tissues (Figure 1K–N). Additionally, we assessed the correlation between RFNG expression and overall survival in CRC patients. Our analysis revealed that higher levels of RFNG were associated with worse prognosis in CRC patients in the SYSU‐FAH cohort and TCGA database (Figure 1O,P). Notably, higher levels of RFNG correlated with worse prognosis at advanced stages (III + IV) but not at early stages (I + II) in patients with CRC (Figure 1Q). In addition, we found that LFNG was highly expressed, whereas MFNG was expressed at low levels in COAD and READ, according to information in the TCGA database (Figure S1Q, Supporting Information). However, the expression of *LFNG* was not correlated with prognosis, whereas high expression of MFNG was associated with poor patient prognosis (Figure S1R, Supporting Information). Taken together, these results demonstrate that RFNG promotes oxaliplatin chemoresistance and that RFNG deficiency sensitizes CRC cells to oxaliplatin treatment. RFNG is highly expressed and serves as a prognostic biomarker for CRC.

### 2.2 RFNG Regulates the Expression of CDKN1A and SLC7A11 to Inhibit Oxaliplatin‐Induced Apoptosis and Ferroptosis

**Figure 2:**
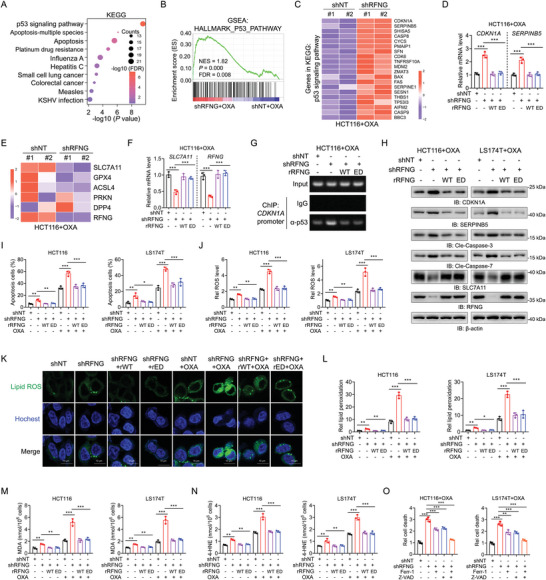
RFNG regulates the expression of CDKN1A and SLC7A11 to inhibit oxaliplatin‐induced apoptosis and ferroptosis. A) RNA‐seq analyses were performed in HCT116 cells stably expressing shNT or shRFNG treated with 20 µm OXA for 12 h. KEGG enrichment analysis of differentially expressed genes is shown. B) GSEA showed that the p53 pathway was enriched among the upregulated pathways in shRFNG cells. C) Heatmap displaying the expression of RFNG knockdown‐regulated p53 pathway genes according to KEGG enrichment analysis. D) HCT116 cells stably expressing shNT, shRFNG, or shRFNG were rescued with rRFNG‐WT or rRFNG‐ED, and qPCR analysis of the mRNA expression of *CDKN1A* and *SERPINB5* was performed after treatment with 20 µm OXA for 12 h. E) Heatmap displaying the expression of indicated genes according to RNA‐seq. F–N) HCT116 and LS174T cells stably expressing shNT, shRFNG, or shRFNG rescued with rRFNG‐WT or rRFNG‐ED were treated with or without 20 µm OXA. F) QPCR analysis of the mRNA expression of *SLC7A11* after treatment for 12 h. G) ChIP PCR analysis of p53 binding at the *CDKN1A* promoters after treatment for 12 h. H) Immunoblotting analysis of the expression of the indicated proteins after treatment for 12 h. I) Flow cytometry analysis of apoptotic cells after treatment for 24 h. J) ROS levels were assessed after treatment for 12 h. K,L) Representative images (K) and relative lipid peroxidation levels (L) were assessed by immunofluorescence using BODIPY C11 staining after treatment for 12 h. M,N) The concentrations of MDA (M) and 4‐NHE (N) were assessed after treatment for 12 h. O) HCT116 cells stably expressing shNT or shRFNG treated with 20 µm OXA plus ferrostatin‐1 (Ferr‐1) or Z‐VAD for 24 h and collected for cell death measurement. *P < 0.05, **P < 0.01, ***P < 0.001 (one‐way ANOVA (D, F, I, J, L, M, N, O)).

To investigate the underlying mechanism by which RFNG promotes chemoresistance to oxaliplatin in CRC cells, we conducted RNA sequencing (RNA‐seq) analysis of RFNG‐depleted HCT116 cells treated with oxaliplatin. Our analysis revealed that the p53 signaling pathway was significantly upregulated in RFNG‐depleted cells, as indicated by the Kyoto Encyclopedia of Genes and Genomes (KEGG) pathway enrichment and gene set enrichment analysis (GSEA) (**Figure** **2**A,B). Heatmaps further demonstrated increased expression of genes from the p53 signaling pathway in RFNG‐knockdown cells (Figure 2C; Figure S2A, Supporting Information). GSEA analysis also demonstrated that RFNG knockdown led to an increase in apoptosis (Figure S2B, Supporting Information). Notably, two well‐established genes in the p53 pathway, *CDKN1A* and *SERPINB5*, which are known to promote tumor cell apoptosis,^[^ ^17^, ^18^ ^]^ exhibited increased expression upon RFNG knockdown, which was rescued by restoration of rRFNG WT or ED expression in RFNG‐depleted cells after oxaliplatin treatment (Figure 2D; Figure S2C, Supporting Information). RFNG overexpression decreased *CDKN1A* and *SERPINB5* expression in HCT116 and LS174T cells (Figure S2D, Supporting Information). In addition, the dual‐luciferase assay results showed that RFNG knockdown significantly increased p53‐luciferase (p53‐luc) activity, whereas restored rRFNG WT or ED expression rescued the increase in p53‐luc activity (Figure S2E, Supporting Information). Accumulating evidence has indicated that p53 plays a role in regulating ferroptosis, which contributes to tumor suppression.^[^ ^19^ ^]^ RNA‐seq analysis revealed that the expression of the ferroptosis‐negative regulatory gene *SLC7A11*, which is known to be transcriptionally inhibited by p53,^[^ ^8^ ^]^ was reduced in RFNG‐depleted cells, while the impact on other p53‐regulated genes^[^ ^20^ ^]^ involved in ferroptosis was relatively minor (Figure 2E). Consistent with these findings, qPCR results demonstrated that RFNG knockdown significantly downregulated the expression of *SLC7A11* and slightly reduced the expression of *GPX4*, while showing minimal effects on *ACSL4*, *PRKN* and *DPP4* expression (Figure S2F,G, Supporting Information). Therefore, our focus is primarily on studying the regulation of SLC7A11 expression by RFNG. RFNG knockdown decreased *SLC7A11* expression and glutathione (GSH) levels, whereas restoration of rRFNG WT or ED expression reversed *SLC7A11* expression and GSH levels (Figure 2F; Figure S2H,I, Supporting Information). Additionally, RFNG overexpression significantly upregulated *SLC7A11* (Figure S2J, Supporting Information). Furthermore, chromatin immunoprecipitation (ChIP) PCR assays revealed that RFNG knockdown led to a significant increase or decrease in the binding of p53 to the promoter regions of *CDKN1A* and *SLC7A11*, respectively, which was markedly reversed by restoration of rRFNG WT or ED expression (Figure 2G; Figure S2K,L, Supporting Information).

As RFNG regulates the expression of *CDKN1A* and *SLC7A11*, we next examined whether RFNG promotes oxaliplatin chemoresistance by inhibiting apoptosis and ferroptosis. We observed a marked increase in the expression of CDKN1A and SERPINB5, which are apoptosis executioners formed via the cleavage of caspase‐3 and caspase‐7, respectively, and a decrease in the expression of SLC7A11 in RFNG‐knockdown cells upon oxaliplatin treatment; these effects were reversed by rRFNG WT or ED overexpression (Figure 2H). Consistent with this, RFNG depletion promoted apoptosis and ferroptosis in HCT116 and LS174T cells, whereas re‐expression of rRFNG WT or ED markedly attenuated apoptosis and ferroptosis, as indicated by the detection of apoptotic cells (Figure 2I) and measurement of ferroptosis indicators, including reactive oxygen species (ROS), lipid peroxidation, malondialdehyde (MDA), and 4‐hydroxynonenal (4‐NHE) levels (Figure 2J–N). Moreover, oxaliplatin‐induced cell death in RFNG‐depleted cells was partially rescued by addition of the ferroptosis inhibitor ferrostatin‐1 (Fer‐1, a lipid peroxidation scavenger) and the apoptosis inhibitor Z‐VAD (Figure 2O). Together, these findings indicate that RFNG regulates CDKN1A and SLC7A11 expression through p53, thereby inhibiting oxaliplatin‐induced apoptosis and ferroptosis.

### 2.3 The Function of RFNG in Promoting Chemoresistance Depends on WT p53

**Figure 3:**
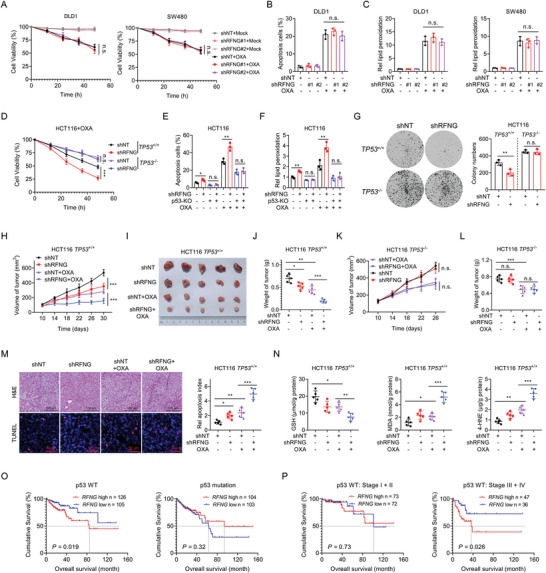
The function of RFNG in promoting chemoresistance depends on WT p53. A–C) DLD1 and SW480 cells stably expressing shNT, shRFNG‐1 or shRFNG‐2 were treated with or without 50 µm OXA. A) Cell viability was assessed after treatment for the indicated times. B) Flow cytometry analysis of apoptotic cells after treatment for 24 h. C) Relative lipid peroxidation levels were assessed after treatment for 12 h. D–F) HCT116 WT (*TP53* ^+/+^) or p53 knockout (*TP53* ^−/−^) cells expressing shNT or shRFNG were treated with or without 20 µm OXA, and cell viability (D), cell apoptosis (E) and relative lipid peroxidation (F) were assessed. G) Colony formation assays were conducted to assess the proliferation of *TP53* ^+/+^ and *TP53* ^−/−^ HCT116 cells stably expressing shNT or shRFNG. H–J) HCT116 *TP53* ^+/+^ cells stably expressing shNT or shRFNG were subcutaneously injected into nude mice, followed by intraperitoneal (i.p.) injection of OXA (7.5 mg kg^−1^) or vehicle (*n* = 5). Statistical analyses of tumor volumes (H), tumor images (I), and tumor weights (J) are shown. K,L) HCT116 *TP53* ^−/−^ cells stably expressing shNT or shRFNG were subcutaneously injected into nude mice, followed by i.p. injection of OXA (7.5 mg kg^−1^) or vehicle (*n* = 5). Statistical analysis of the tumor volumes (K) and tumor weights (L) are shown. M) Hematoxylin‐eosin (H&E) and TUNEL staining (left) and quantification of the apoptotic index (TUNEL staining) (right) in xenograft tumors from (H). N) GSH, MDA, and 4‐HNE levels were assessed in xenograft tumors from (H). O) Kaplan‒Meier analysis of overall survival according to RFNG expression in p53 WT or p53 mutant CRC patient samples from the TCGA COAD database. P) Kaplan‒Meier analysis of overall survival according to RFNG expression in stage I+II (left) or stage III+IV (right) p53 WT CRC patient samples from the TCGA COAD database. *P < 0.05, **P < 0.01, ***P < 0.001, n.s. = non‐significant (two‐way ANOVA (A, D, H, K), one‐way ANOVA (B, C, E, F, G, J, L, M, N), or log‐rank test (O, P)).

Next, we sought to determine whether RFNG promotes chemoresistance in a p53‐dependent manner. We knocked down RFNG in the p53 mutant CRC cell lines DLD1 and SW480 (Figure S3A, Supporting Information). Interestingly, RFNG depletion did not affect cell survival, apoptosis, or ferroptosis induced by oxaliplatin treatment in p53 mutant cells (**Figure** **3**A–C; Figure S3B,C, Supporting Information). Additionally, RFNG knockdown did not affect the proliferation of these cells (Figure S3D,E, Supporting Information). To confirm the role of p53 in mediating the effects of RFNG, we knocked down RFNG in *TP53* ^+/+^ (which contains functional p53) and *TP53* ^−/−^ (p53‐defective) HCT116 cells (Figure S3F, Supporting Information). As expected, RFNG depletion significantly decreased cell survival and increased apoptosis and ferroptosis in the *TP53* ^+/+^ HCT116 cells. However, in *TP53* ^−/−^ HCT116 cells, RFNG knockdown had no effect on these cellular processes (Figure 3D–F; Figure S3G, Supporting Information). In addition, RFNG knockdown inhibited the proliferation of *TP53* ^+/+^ HCT116 cells, but not that of *TP53* ^−/−^ HCT116 cells (Figure 3G; Figure S3H, Supporting Information).

To further elucidate the role of RFNG in the promotion of chemoresistance in vivo, we established a xenograft mouse model. Our results demonstrated that knockdown of RFNG in *TP53* ^+/+^ HCT116 cells inhibited the growth of tumor cells in vivo and significantly enhanced the killing effect of oxaliplatin (Figure 3H–J). However, RFNG knockdown in *TP53* ^−/−^ HCT116 cells had little effect on tumor cell growth or oxaliplatin chemoresistance in xenograft models (Figure 3K,L; Figure S3I, Supporting Information). Next, we measured the levels of apoptosis and ferroptosis in the xenografts derived from *TP53* ^+/+^ HCT116 cells. We found that RFNG knockdown significantly increased the expression of *CDKN1A* and *SERPINB5* and decreased the expression of *SLC7A11* upon oxaliplatin treatment (Figure S3J, Supporting Information). Consistent with these findings, RFNG depletion enhanced oxaliplatin chemotherapy‐induced tumor cell apoptosis and ferroptosis, as determined by increased terminal deoxynucleotidyl transferase dUTP nick‐end labeling (TUNEL) staining, decreased GSH levels, and elevated MDA and 4‐NHE levels (Figure 3M,N). Furthermore, we analyzed the clinical relevance of RFNG expression and p53 mutation status in CRC patients using data from TCGA database. We found that patients with high RFNG expression had a worse prognosis in the p53 WT group, but not in the p53 mutant group (Figure 3O). Notably, higher RFNG levels were associated with worse prognosis in advanced‐stage patients, but not in early‐stage patients in the p53 wild‐type group (Figure 3P). Taken together, these findings suggest that the function of RFNG in promoting chemoresistance depends on the WT p53.

### 2.4 RFNG Undergoes Phosphorylation at the S255 Residue and Nuclear Translocation upon Oxaliplatin Treatment

**Figure 4:**
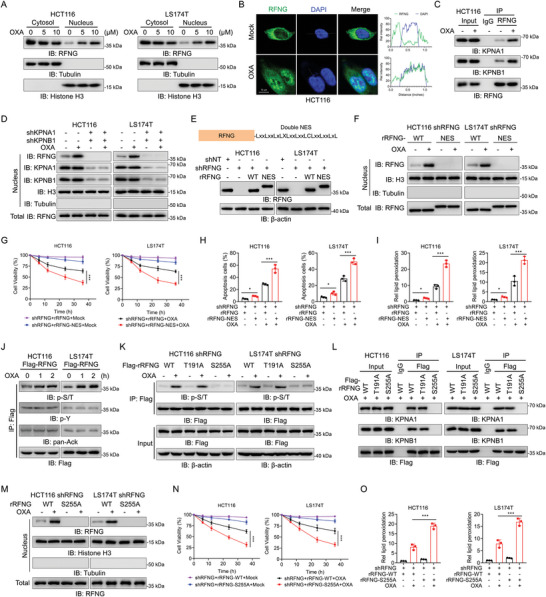
RFNG undergoes phosphorylation at the S255 residue and nuclear translocation upon oxaliplatin treatment. A) HCT116 and LS174T cells were treated with 5 or 10 µm OXA for 1 h, and cytosolic and nuclear fractions were collected for immunoblotting analysis. B) HCT116 cells were treated with or without 10 µm OXA for 1 h, and then immunofluorescence (IF) staining was performed. RFNG localization was indicated by an anti‐RFNG antibody, and nuclei were labeled with DAPI (left). Signal intensities and distances were quantified (right). Scale bar, 10 µm. C) HCT116 cells were treated with or without 10 µm OXA for 1 h, after which coimmunoprecipitation (co‐IP) was performed. D) HCT116 and LS174T cells stably expressing shNT and shKPNA1/shKPNB1 were treated with or without 10 µm OXA for 1 h, and cellular fractions were collected as indicated. Immunoblotting analysis was performed with indicated antibodies. E) Construction of RFNG tagged with a double nuclear export sequence (NES) at its C‐terminus (RFNG‐NES) (upper). Immunoblotting analysis of RFNG expression in HCT116 and LS174T cells stably expressing shNT, shRFNG, or shRFNG rescued with rRFNG‐WT or rRFNG‐NES (lower). F) RFNG‐knockdown HCT116 and LS174T cells stably expressing rRFNG‐WT or rRFNG‐NES were treated with or without 10 µm OXA for 1 h, and the cellular fractions were collected as indicated. Immunoblotting analysis was performed with indicated antibodies. G–I) RFNG‐knockdown HCT116 and LS174T cells stably expressing rRFNG‐WT or rRFNG‐NES were treated with or without 20 µm OXA, and cell viability (G), apoptosis (H) and relative lipid peroxidation (I) were assessed. J) HCT116 and LS174T cells stably expressing Flag‐RFNG were treated with 10 µm OXA for the indicated times, and immunoblotting analysis was performed. p‐S/T, serine/threonine phosphorylation; p‐Y, tyrosine phosphorylation; pan‐Ack, panlysine acetylation. K) RFNG‐knockdown HCT116 and LS174T cells stably expressing rRFNG‐WT, rRFNG‐T191A or rRFNG‐S255A were treated with or without 10 µm OXA for 1 h, and co‐IP was performed. L) RFNG‐knockdown HCT116 and LS174T cells stably expressing rRFNG‐WT, rRFNG‐T191A or rRFNG‐S255A were treated with 10 µm OXA for 1 h, and co‐IP was performed. M) RFNG‐knockdown HCT116 and LS174T cells stably expressing rRFNG‐WT or rRFNG‐S255A were treated with or without 10 µm OXA for 1 h, and the cellular fractions were collected as indicated. Immunoblotting analysis was performed with indicated antibodies. N,O) RFNG‐knockdown HCT116 and LS174T cells stably expressing rRFNG‐WT or rRFNG‐S255A were treated with or without 20 µm OXA, and cell viability (N) and relative lipid peroxidation (O) were assessed. *P < 0.05, ***P < 0.001 (two‐way ANOVA (G, N) or one‐way ANOVA (H, I, O)).

Next, we studied how RFNG regulates p53 activity under oxaliplatin stimulation. Because p53 is primarily localized in the nucleus,^[^ ^21^ ^]^ we first investigated the intracellular localization of RFNG. We performed cell fractionation experiments and found that RFNG was predominantly present in the cytoplasm but translocated to the nucleus after oxaliplatin treatment in HCT116 and LS174T cells (**Figure** **4**A). This observation was further confirmed by immunofluorescence staining, which showed a noticeable increase in nuclear accumulation of RFNG following oxaliplatin treatment (Figure 4B; Figure S4A, Supporting Information). Additionally, RFNG was associated with KPNA1 (also known as importin α1) and KPNB1 (also known as importin β1), which are members of the importin family, specifically under oxaliplatin treatment conditions (Figure 4C; Figure S4B, Supporting Information), further suggesting an importin‐dependent mechanism of RFNG nuclear translocation. Fractionation analysis revealed that silencing KPNA1 and KPNB1 inhibited oxaliplatin‐induced nuclear accumulation of RFNG (Figure 4D). These findings led us to speculate that nuclear localization is a prerequisite for RFNG to promote chemoresistance. To test this hypothesis, we modified RFNG by adding a double nuclear export sequence (NES) to its C‐terminus (Figure 4E). RFNG‐NES was successfully localized to the cytosol following oxaliplatin treatment (Figure 4F). Moreover, we found that compared with rRFNG WT, restoration of rRFNG NES expression in RFNG‐depleted cells significantly inhibited cell survival and increased apoptosis and ferroptosis in HCT116 and LS174T cells (Figure 4G–I; Figure S4C, Supporting Information).

To investigate how upstream factors regulate the nuclear localization of RFNG, we examined posttranslational modifications of RFNG. We found that upon oxaliplatin stimulation, the level of serine/threonine phosphorylation (p‐S/T) of RFNG significantly increased, whereas tyrosine phosphorylation (p‐Y) and panlysine acetylation (pan‐Ack) remained unchanged (Figure 4J). Using the PhosphoSitePlus database (https://www.phosphosite.org/homeAction), we identified two potential serine/threonine phosphorylation sites in RFNG, T191 and S255 (Figure S4D, Supporting Information). These two residues were individually mutated to alanine (A). Restoration of the rRFNG S255A mutant dramatically inhibited RFNG phosphorylation upon oxaliplatin treatment, whereas T191A hardly affected this phosphorylation, suggesting that S255 was the phosphorylated residue (Figure 4K). Furthermore, the RFNG S255A mutant was unable to bind to KPNA1 or KPNB1 (Figure 4L), and failed to translocate to the nucleus (Figure 4M). QPCR and immunoblotting analysis revealed that the expression of the rRFNG S255A mutant promoted *CDKN1A* expression and decreased *SLC7A11* expression (Figure S4E,F, Supporting Information). Consistent with this finding, restoration of the rRFNG S255A mutant in RFNG‐knockdown cells resulted in decreased cell survival and increased apoptosis and ferroptosis compared to the restoration of rRFNG WT (Figure 4N,O; Figure S4G,H, Supporting Information). Taken together, these results indicate that RFNG undergoes phosphorylation at the S255 residue and subsequent nuclear translocation upon oxaliplatin treatment, which is essential for the RFNG‐mediated promotion of chemoresistance.

### 2.5 ERK Phosphorylates RFNG to Promote its Translocation

**Figure 5:**
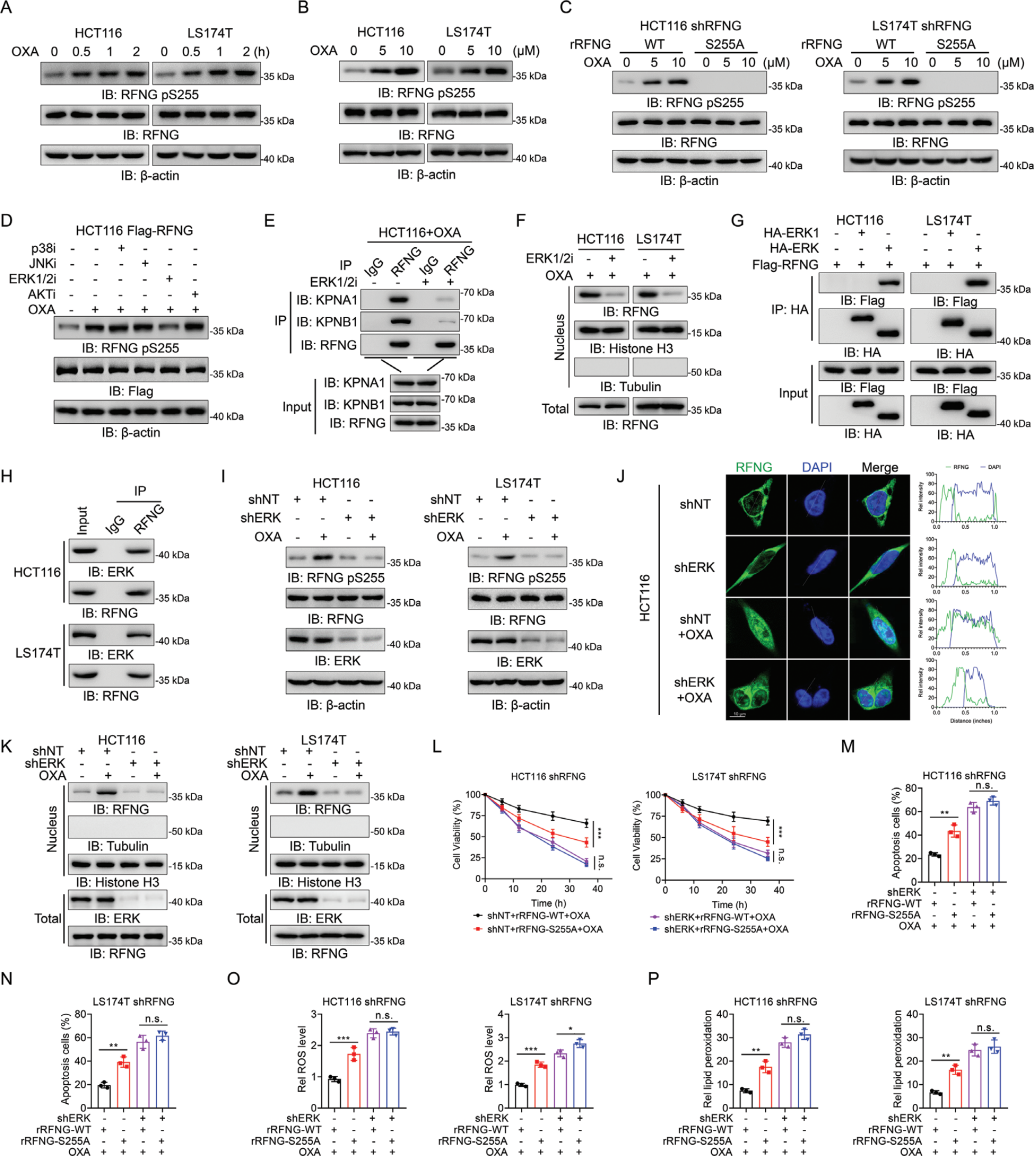
ERK phosphorylates RFNG to promote its translocation. A) HCT116 and LS174T cells were treated with 10 µm OXA for the indicated times, and immunoblotting analysis was performed. B) HCT116 and LS174T cells were treated with 5 µm or 10 µm OXA for 1 h, after which immunoblotting analysis was performed. C) RFNG‐knockdown HCT116 and LS174T cells stably expressing rRFNG‐WT or rRFNG‐S255A were treated with 5 µm or 10 µm OXA for 1 h, after which immunoblotting analysis was performed. D) HCT116 cells stably expressing Flag‐RFNG were treated with or without the indicated inhibitors for 12 h, followed by treatment with 10 µm OXA for 1 h. Immunoblotting analysis was subsequently performed. E,F) HCT116 or LS174T cells were treated with or without ERK1/2 inhibitors (ERK1/2i) for 12 h, followed by 10 µm OXA for 1 h. Co‐IP (E) and immunoblotting (F) were performed. G) HCT116 and LS174T cells stably expressing Flag‐RFNG were transfected with HA‐vector, ERK1, or ERK2 for 24 h, followed by 10 µm OXA treatment for 1 h. Co‐IP was subsequently performed. H) HCT116 and LS174T cells were treated with 10 µm OXA for 1 h, and co‐IP was performed. I–K) HCT116 and LS174T cells stably expressing shNT and shERK were treated with or without 10 µm OXA for 1 h. Immunoblotting (I), IF (J) and cellular fraction analysis (K) were performed. Scale bar, 10 µm. L–P) RFNG‐depleted HCT116 and LS174T cells stably expressing shNT or shERK were infected with rRFNG‐WT or rRFNG‐S255A and treated with 20 µm OXA. Cell viability (L), apoptosis (M, N), relative ROS levels (O) and relative lipid peroxidation (P) were assessed. *P < 0.05, **P < 0.01, ***P < 0.001, n.s. = non‐significant (two‐way ANOVA (L), or one‐way ANOVA (M, N, O, P)).

To identify the kinase responsible for RFNG S255 phosphorylation (pS255), we developed an antibody specific to RFNG pS255. We validated the specificity of the antibody and found that RFNG depletion in cells rescued by the rRFNG S255A mutation abrogated RFNG pS255 (Figure S5A, Supporting Information). Furthermore, we observed that the level of RFNG pS255 increased with increasing duration of oxaliplatin stimulation (**Figure** **5**A). Additionally, oxaliplatin stimulation induced RFNG pS255 expression in a dose‐dependent manner (Figure 5B). However, the restoration of the rRFNG S255A mutant in RFNG‐depleted cells abolished RFNG pS255 (Figure 5C). To further investigate the signaling pathway associated with RFNG pS255, we treated the cells with inhibitors targeting key kinases activated by oxaliplatin treatment.^[^ ^22^, ^23^, ^24^ ^]^ The results showed that inhibition of ERK1/2 attenuated oxaliplatin‐induced RFNG pS255 expression in HCT116 and LS174T cells (Figure 5D; Figure S5B, Supporting Information). The RFNG S255 residue, which is conserved in mammalian species, is located within the “SP” site as the MAPK phosphorylation consensus site (Figure S5C, Supporting Information). In addition, after treatment with ERK1/2 inhibitors, RFNG exhibited reduced binding to KPNA1 and KPNB1 in HCT116 and LS174T cells (Figure 5E; Figure S5D, Supporting Information) and its translocation to the nucleus was impaired (Figure 5F). These results indicate that the activity of ERK1/2, a key component of the MAPK signaling pathway, is crucial for RFNG pS255 and its subsequent translocation.

We further investigated the interaction between RFNG and ERK1/2 and found that RFNG specifically binds to ERK (also known as ERK2) rather than to ERK1 (Figure 5G), which was confirmed by endogenous co‐immunoprecipitation assays (Figure 5H). Furthermore, we found that oxaliplatin treatment promoted ERK phosphorylation in HCT116 and LS174T cells (Figure S5E, Supporting Information). In vitro kinase assays demonstrated that ERK directly phosphorylated RFNG at S255 residue (Figure S5F, Supporting Information). Moreover, ERK depletion inhibited oxaliplatin‐induced RFNG pS255 expression and nuclear translocation (Figure 5J,K; Figure S5G, Supporting Information), indicating that ERK was responsible for promoting RFNG pS255. Reconstitution with the rRFNG S255A mutant also did not affect the expression of *CDKN1A* or *SLC7A11* in ERK knockdown cells (Figure S5H,I, Supporting Information). Correspondingly, in control cells, expression of the recombinant rRFNG S255A mutant resulted in a lower cell survival rate, greater cell apoptosis, and greater ferroptosis than did expression of the recombinant rRFNG WT, while in ERK‐knockdown cells, there was little difference in the effects of the recombinant rRFNGS255A mutant or WT on cell apoptosis and ferroptosis (Figure 5L–P). These results suggest that the ERK‐mediated phosphorylation of RFNG at S255 is crucial for its functional effects. Overall, our findings highlight the essential role of ERK in RFNG phosphorylation and nuclear translocation.

### 2.6 RFNG pS255 Binds to p53 and Inhibits its Phosphorylation

**Figure 6:**
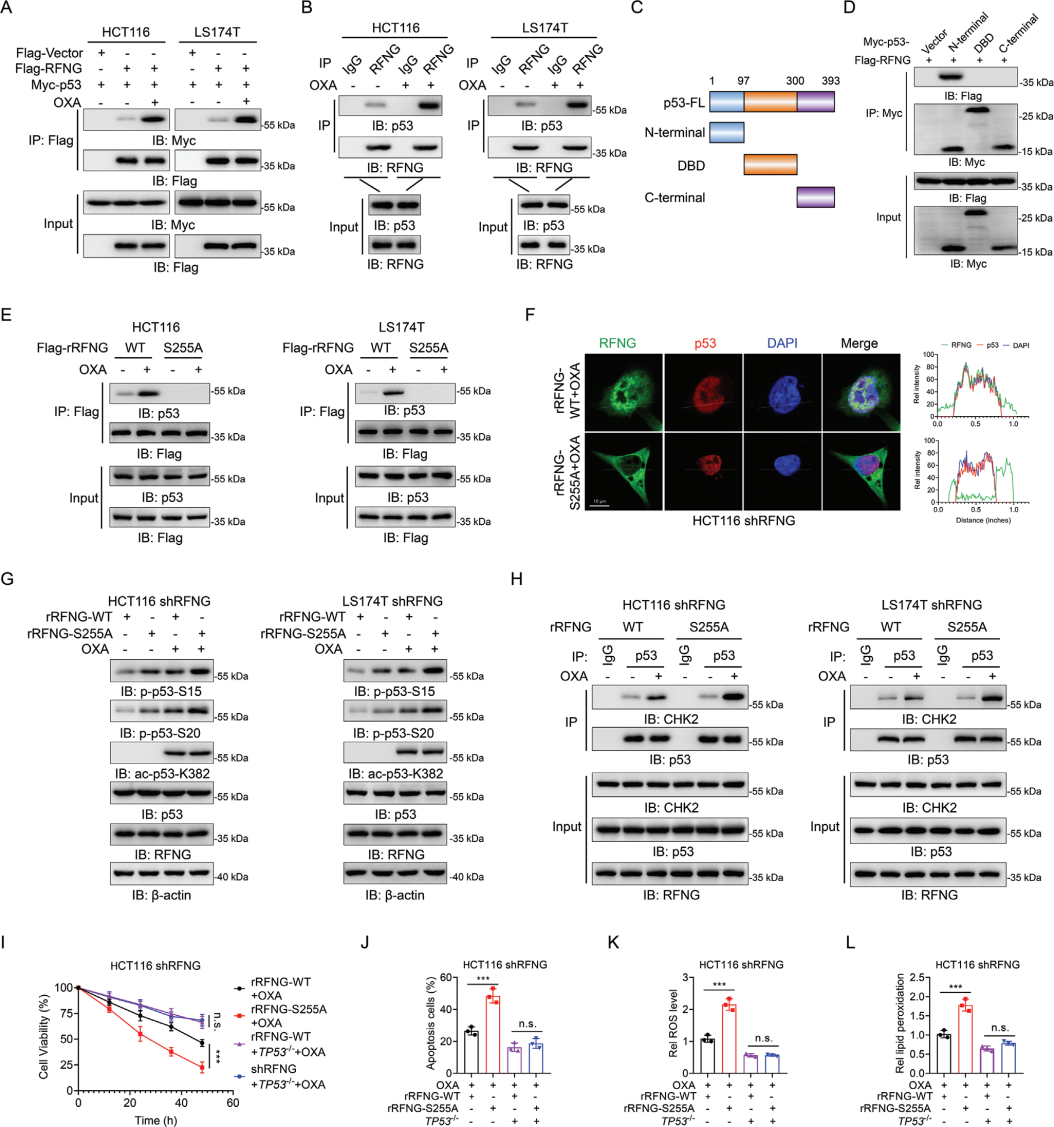
RFNG pS255 binds to p53 and inhibits its phosphorylation. A) HCT116 and LS174T cells stably expressing Flag‐vector and RFNG were transfected with Myc‐p53 and then treated with or without 10 µm OXA for 1 h. Co‐IP was subsequently performed. B) HCT116 and LS174T cells were treated with or without 10 µm OXA for 1 h, and co‐IP was performed. C) Schematic diagram of p53 and its truncation mutants. D) HCT116 cells stably expressing Flag‐RFNG were transfected with Myc‐p53 or its truncations, and co‐IP was performed. E) HCT116 or LS174T cells stably expressing Flag‐rRFNG‐WT or rRFNG‐S255A were treated with or without 10 µm OXA for 1 h, co‐IP was performed. F) RFNG‐knockdown HCT116 or LS174T cells stably expressing rRFNG‐WT or rRFNG‐S255A were treated with 10 µm OXA for 1 h, IF analysis was performed. Scale bar, 10 µm. G,H) RFNG‐knockdown HCT116 or LS174T cells stably expressing rRFNG‐WT or rRFNG‐S255A were treated with or without 10 µm OXA for 1 h, immunoblotting analyses (G) and Co‐IP (H) were performed. I–L) RFNG‐depleted HCT116 *TP53* ^+/+^ or *TP53* ^−/−^ cells were infected with rRFNG‐WT or rRFNG‐S255A and then treated with 20 µm OXA. Cell viability (I), apoptosis (J), relative ROS levels (K) and relative lipid peroxidation (L) were assessed. ***P < 0.001, n.s. = non‐significant (two‐way ANOVA (I) or one‐way ANOVA (J, K, L)).

RFNG undergoes dynamic cellular localization in response to oxaliplatin, suggesting a potential role of this protein in modulating p53 activity in the nucleus. We first investigated the interaction between RFNG and p53 in response to oxaliplatin stimulation. The results revealed that the binding of RFNG to p53 notably increased upon oxaliplatin stimulation (**Figure** **6**A). This interaction was also observed between endogenous RFNG and p53 (Figure 6B). Both RFNG WT and ED were able to bind to p53 in HCT116 cells (Figure S6A, Supporting Information), but RFNG did not bind to mutant p53 in DLD1 or SW480 cells (Figure S6B, Supporting Information). By generating p53 truncation mutants, we determined that the N‐terminus of p53 was responsible for its interaction with RFNG (Figure 6C,D). Moreover, oxaliplatin significantly enhanced the binding of WT RFNG to p53, whereas the RFNG S255A mutant failed to bind to p53 (Figure 6E). Immunofluorescence analysis further confirmed that RFNG WT and p53 co‐localized in the nucleus, whereas the RFNG S255A mutant remained in the cytoplasm and did not co‐localize with p53 upon oxaliplatin treatment (Figure 6F; Figure S6C, Supporting Information). These data demonstrated that oxaliplatin stimulation promotes RFNG translocation to the nucleus, thereby enhancing its interaction with p53.

To understand how RFNG represses p53 activity through its interaction, we examined the phosphorylation, acetylation, and total levels of p53, as these parameters are known to play crucial roles in controlling p53 function.^[^ ^25^ ^]^ RFNG overexpression had minimal effects on p53 mRNA and protein expression as well as p53 acetylation, but significantly inhibited p53 phosphorylation (Figure S6D,E, Supporting Information). Furthermore, compared with RFNG WT, reconstitution of the RFNG S255A mutant promoted p53 phosphorylation but did not affect p53 acetylation or total protein levels (Figure 6G). To further elucidate the mechanism by which RFNG inhibited p53 phosphorylation, we investigated whether RFNG suppresses kinase‐mediated phosphorylation of p53. Individual knockdown of kinases known to phosphorylate p53 under DNA damage^[^ ^5^ ^]^ revealed that only CHK2 knockdown significantly attenuated the increase in p53 phosphorylation upon RFNG depletion (Figure S6F,G, Supporting Information), indicating that RFNG acts by modulating CHK2‐mediated phosphorylation of p53. Chemotherapy‐induced DNA damage usually triggers the activation of CHK2, which subsequently leads to the phosphorylation of p53.^[^ ^26^ ^]^ Our findings showed that RFNG did not interfere with CHK2 phosphorylation or expression (Figure S6H, Supporting Information), nor did it physically interact with CHK2 (Figure S6I, Supporting Information). We speculate that RFNG may interfere with the binding of CHK2 to p53. Our results demonstrated that the interaction between CHK2 and p53 was significantly enhanced in RFNG‐depleted cells compared to control cells (Figure S6J, Supporting Information). This observation was supported by the stronger interaction between CHK2 and p53 in rRFNG S255A cells compared to rRFNG WT cells (Figure 6H). These results suggest that RFNG competitively inhibits the association of CHK2 with p53, thereby preventing p53 phosphorylation and inhibiting its activity.

To confirm the role of RFNG in p53‐dependent processes, we restored the expression of rRFNG WT or S255A mutant in RFNG‐depleted HCT116 cells with or without p53 knockout (Figure S6K, Supporting Information). Restoration of the rRFNG S255A mutant in *TP53* ^+/+^ cells regulated the expression of *CDKN1A*, *SERPINB5* and *SLC7A11* but had little effect in *TP53* ^−/−^ cells (Figure S6L, Supporting Information). Accordingly, restoration of the rRFNG S255A mutant in *TP53* ^+/+^ cells resulted in decreased cell survival and increased apoptosis and ferroptosis compared to that of rRFNG WT; however, restoration of either the rRFNG S255A mutant or WT in *TP53* ^−/−^ cells had little effect on the induction of apoptosis or ferroptosis (Figure 6I–L). Taken together, these results demonstrated that RFNG pS255 binds to p53 and inhibits its phosphorylation, thereby promoting chemoresistance.

### 2.7 RFNG pS255 Levels are Negatively Correlated with the Efficacy of Chemotherapy and the Prognosis of CRC Patients

**Figure 7:**
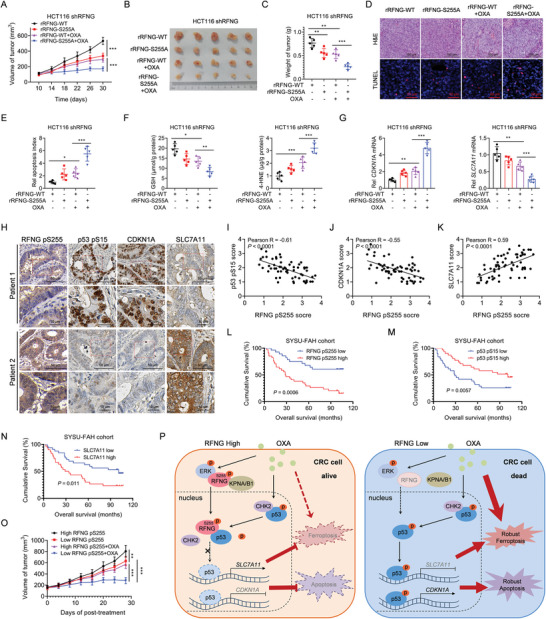
RFNG pS255 levels are negatively correlated with the efficacy of chemotherapy and the prognosis of CRC patients. A–G) RFNG‐knockdown HCT116 cells stably expressing rRFNG‐WT or S255A were subcutaneously injected into nude mice, followed by i.p. injection of OXA (7.5 mg kg^−1^) or vehicle (*n* = 5). A–C) Statistical analysis of tumor volumes (A), tumor images (B) and tumor weights (C) are shown. D, E) H&E and TUNEL staining (D) and quantification of the apoptotic index (TUNEL staining) (E) in xenograft tumors. F) The GSH and 4‐HNE levels were assessed in xenograft tumors. G) QPCR analysis of the mRNA expression of *CDKN1A* and *SLC7A11* in the indicated xenograft tumors. H) IHC staining of human CRC clinical samples using antibodies against RFNG pS255, p53 pS15, CDKN1A, and SLC7A11. Representative images are shown. I–K) Correlation analysis of IHC staining levels between RFNG pS255 and p53 pS15 (I), RFNG pS255 and CDKN1A (J), and RFNG pS255 and SLC7A11 (K) in human CRC samples. L–N) Kaplan‒Meier analysis of overall survival according to RFNG pS255 (L), p53 pS15 (M), and SLC7A11 protein (N) levels in human CRC samples. O) Oxaliplatin (7.5 mg kg^−1^) treatment was tested in patient‐derived xenograft (PDX) models. Human CRC samples were collected for PDX preparation based on the histological expression of high or low levels of RFNG pS255. The results of the statistical analysis of the tumor volumes are shown. P) A proposed model of the mechanism by which RFNG is phosphorylated and translocated into the nucleus to restrain oxaliplatin‐induced apoptosis and ferroptosis in CRC cells. *P < 0.05, **P < 0.01, ***P < 0.001 (two‐way ANOVA (A, O), one‐way ANOVA (C, E, F, G), or log‐rank test (L, M, N)).

We further investigated the correlation between RFNG pS255 level and chemotherapeutic efficacy in vivo. We found that compared to tumors with rRFNG WT, tumors with rRFNG S255A exhibited a notable response to oxaliplatin chemotherapy, as indicated by slower tumor growth and lower tumor volume and weight (**Figure** **7**A–C). Consistent with these findings, rRFNG S255A tumors treated with oxaliplatin exhibited increased apoptosis and ferroptosis rates (Figure 7D–F; Figure S7A, Supporting Information). Furthermore, compared with those in rRFNG WT tumors, the expression of *CDKN1A* and *SERPINB5* was significantly upregulated, whereas the expression of *SLC7A11* was downregulated in rRFNG S255A tumors treated with oxaliplatin (Figure 7G; Figure S7B, Supporting Information).

To further explore the clinical relevance of RFNG pS255‐regulated p53 phosphorylation, as well as CDKN1A and SLC7A11 protein levels, we collected samples from CRC patients with p53 WT receiving oxaliplatin‐based chemotherapy. We analyzed the levels of RFNG S255 and p53 S15 phosphorylation, and CDKN1A and SLC7A11 protein expression in these tissues. The IHC staining results revealed a strong negative correlation between RFNG pS255 levels and p53 pS15 and CDKN1A protein levels, whereas a positive correlation was observed with SLC7A11 protein levels (Figure 7H–K). Based on the IHC staining results, we categorized RFNG pS255, p53 pS15, CDKN1A, and SLC7A11 samples into high‐ or low‐expression groups. Overall survival analysis demonstrated that higher levels of RFNG pS255 and SLC7A11 predicted a poor prognosis, whereas higher levels of p53 pS15 and CDKN1A were associated with a better prognosis (Figure 7L–N; Figure S7C, Supporting Information). In an oxaliplatin treatment test using patient‐derived xenograft (PDX) models with p53 WT, the group with lower levels of RFNG pS255 exhibited significant sensitivity to oxaliplatin chemotherapy (Figure 7O; Figure S7D, Supporting Information), suggesting that an appropriate context was necessary for selecting oxaliplatin treatment in CRC patients. In summary, these results demonstrate that RFNG pS255 level is negatively correlated with chemotherapy efficacy and prognosis of CRC patients with CRC.

## 3 Discussion

Platinum‐based drugs such as cisplatin, carboplatin, and oxaliplatin are commonly used in chemotherapy to treat cancer. However, the development of resistance to platinum‐based drugs, such as oxaliplatin, significantly limits their effectiveness in clinical applications.^[^ ^27^ ^]^ This resistance often occurs because of the adaptive tolerance of tumor cells to cell death mechanisms, including apoptosis and ferroptosis.^[^ ^28^ ^]^ Therefore, it is crucial to investigate the molecular mechanisms underlying tumor cell resistance to oxaliplatin‐induced apoptosis and ferroptosis to develop effective strategies to overcome chemotherapy resistance. In this study, we discovered that, under oxaliplatin treatment, CRC cells phosphorylate RFNG at the Ser255 residue through the MAPK kinase ERK, leading to the binding of RFNG to nuclear importin proteins and its subsequent entry into the nucleus. Once in the nucleus, RFNG interacts with p53 and inhibits its activity of p53. RFNG played an important role in the resistance of CRC cells to apoptosis and ferroptosis in response to oxaliplatin chemotherapy (Figure 7P). Our findings highlight the RFNG‐mediated defense mechanism in promoting chemotherapy resistance in CRC cells and suggest a therapeutic approach that targets apoptosis and ferroptosis for the treatment of CRC.

Fringe proteins have been implicated in the regulation of the Notch signaling pathway that drives cancer initiation and tumorigenesis.^[^ ^12^, ^29^, ^30^ ^]^ LFNG has been extensively studied among human Fringes, whereas its homologs, MFNG and RFNG, are relatively understudied. LFNG is the dominant mammalian fringe gene because of its relatively high catalytic efficiency.^[^ ^31^ ^]^ Studies have shown that the elimination of LFNG or its mutations can lead to developmental defects in mice, such as shorter tails, impaired ribs,^[^ ^32^ ^]^ somite formation defects,^[^ ^33^ ^]^ and subfertile phenotypes.^[^ ^34^ ^]^ Further investigation of these fringe members is necessary to determine their clinical significance. Previous studies on fringes have focused mainly on their enzymatic activity and their contribution to tumor progression. However, given the redundancy among fringe members and the lower catalytic efficiency of RFNG, non‐enzymatic regulation may also play a significant role in tumor survival and progression. In this study, we made a significant discovery regarding the enzymatic activity‐independent non‐canonical function of RFNG, revealing its crucial role in promoting chemoresistance in CRC cells. Our study highlights the potential of targeting RFNG as a promising strategy to overcome chemoresistance in CRC and provides exciting prospects for the development of novel drugs to treat CRC.

In recent years, significant progress has been made in the understanding of the mechanisms of ferroptosis, a form of iron‐dependent cell death. There are three main iron‐induced death defense systems: the SLC7A11‐GSH‐GPX4, NAD(P)H‐FSP1‐CoQ10, and GCH1‐BH_4_/BH_2_ system.^[^ ^35^ ^]^ The cystine/glutamate antiporter SLC7A11 (also known as xCT) is often overexpressed in various human cancers, and studies have shown that its overexpression promotes tumor growth by suppressing ferroptosis.^[^ ^36^, ^37^ ^]^ In our study, we discovered that high‐level phosphorylation of RFNG in cells leads to its nuclear localization, where it inhibits p53 and promotes the expression of SLC7A11 during oxaliplatin chemotherapy. This, in turn, reduces reactive oxygen species (ROS) levels and inhibits ferroptosis. Conversely, cells with low RFNG phosphorylation levels exhibited increased ferroptosis. Therefore, we propose the use of erastin, a SLC7A11 inhibitor, to block the RFNG‐p53‐SLC7A11 axis and enhance the sensitivity of CRC cells to oxaliplatin by promoting ferroptosis. This strategy holds promise for targeting advanced CRC with highly activated RFNG. Additionally, RFNG inhibits oxaliplatin‐induced apoptosis, thus conferring resistance to oxaliplatin chemotherapy. These findings suggest that RFNG phosphorylation may serve as a potential biomarker for predicting the efficacy of oxaliplatin chemotherapy.

Dysregulation of MAPK and p53 signaling has been implicated in tumor progression.^[^ ^38^, ^39^ ^]^ Our previous research showed that abnormal activation of MAPK signaling promotes the proliferation of tumor cells,^[^ ^40^ ^]^ whereas inhibition of p53 activity promotes the progression and chemoresistance of CRC cells.^[^ ^41^ ^]^ However, the crosstalk between these signaling pathways in oxaliplatin chemotherapy for CRC remains poorly understood. In the present study, we investigated the interactions between MAPK and p53 signaling in chemoresistance of CRC cells by focusing on the role of the RFNG protein. Upon oxaliplatin stimulation, the ERK kinase of the MAPK signaling pathway phosphorylates RFNG at the S255 residue, leading to its binding to the importin protein and subsequent translocation into the nucleus, where it inhibits p53 activity. Notably, phosphorylation of RFNG at S255 is essential for its nuclear translocation, inhibition of p53 signaling, and suppression of oxaliplatin‐induced apoptosis and ferroptosis in CRC cells. Our findings highlight the crosstalk between the MAPK and p53 signaling pathways through RFNG, which mediates oxaliplatin resistance in CRC cells. Furthermore, our study provides guidance for oxaliplatin treatment of patients with CRC. Oxaliplatin may induce nuclear accumulation of RFNG in CRC cells with high ERK activity, and in CRC cells with low ERK activity, oxaliplatin may not be necessary to elicit the function of nuclear RFNG. Understanding these molecular mechanisms offers valuable insights for the development of new therapeutic strategies for CRC management.

## 4 Experimental Section

### Cell Lines

The HCT116, LS174T, RKO, LoVo, DLD1, SW480, and HEK293T cell lines were obtained from the American Type Culture Collection (ATCC). All cell lines were cultured at 37 °C in a 5% CO_2_ atmosphere in DMEM or RPMI 1640 medium supplemented with 10% fetal bovine serum (Corning, #35‐079‐CV) and penicillin/streptomycin (Thermo Fisher Scientific, #15 070 063). All cell lines were authenticated using the short tandem repeat method and tested negative for Mycoplasma.

### Plasmids and Reagents

The expression plasmids for RFNG, rRFNG WT, rRFNG ED, rRFNG T191A, and rRFNG S255A were constructed in the pCDH‐EF1‐MCS‐T2A‐Puro vector using the ClonExpress II One Step Cloning Kit (Vazyme, #C112). For transient transfection, Lipofectamine 2000 (Thermo Fisher Scientific, #11 668 019) was used according to the manufacturer's protocol. Oxaliplatin (#S1224), Ferrostatin‐1 (#S7243), Z‐VAD (#S7023), the p38 inhibitor VX‐702 (#S6005), the JNK inhibitor JNK‐IN‐8 (#S4901), the ERK1/2 inhibitor SCH772984 (#S7101) and the AKT inhibitor MK‐2206 (#S1078) were obtained from Selleck. Puromycin (#P8833), polybrene (#TR‐1003) and trypan blue solution (#T8154) were purchased from Sigma Aldrich.

### SiRNA or shRNA Interference and CRISPR/Cas9 Knockout

The siRNAs corresponding to the target sequences were synthesized by GenePharma (Suzhou, China). shRNAs targeting RFNG and ERK were inserted into the pLKO.1 vector. The sgRNAs targeting p53 were cloned and inserted into the lentiCRISPR‐v2 vector. Then, the expression plasmids, the lentiviral packaging plasmid psPAX.2 (Addgene, #12 260) and the envelope plasmid pMD2.G (Addgene, #12 259) were cotransfected into HEK293T cells. After 48 h, the lentiviruses were used to infect cells, and the cells were then screened with puromycin for 3 days. The sequences of the siRNAs, shRNAs, and sgRNAs used are listed in Table S1 (Supporting Information).

### Cell Viability Assay

Cells were seeded in 12‐well plates and treated with or without oxaliplatin for the indicated times. Cell viability was assessed by cell counting after staining with trypan blue.

### Cell Proliferation Assay

For cell growth curves, cell growth was measured using the cell count method. For the colony formation assay, cells were seeded into six‐well plates and cultured for 7 to 10 days. The cells were then fixed with 4% paraformaldehyde and stained with crystal violet staining solution. Colonies were visualized and counted.

### Cell Apoptosis Detection

Cells were treated with oxaliplatin for the indicated times and then harvested. Cell apoptosis was measured with an Annexin V‐FITC/PI Apoptosis Detection Kit (Vazyme, #A211) according to the manufacturer's instructions.

### Determination of Lipid Peroxidation

Cells were treated with oxaliplatin for the indicated time and then stained with the lipid peroxidation sensor BODIPY 581/591 C11 (5 µm; Thermo Fisher Scientific, #D3861) at 37 °C for 20 min. For immunofluorescence analysis, the cells were then stained with Hoechst (Thermo Fisher Scientific, #62 249) for 10 min. The levels of lipid peroxidation were analyzed by immunofluorescence microscopy.

### Reactive Oxygen Species (ROS) Detection

ROS levels in cells and tissues were measured using the Reactive Oxygen Species (ROS) Fluorometric Assay Kit (Elabscience, #E‐BC‐K138‐F) according to the manufacturer's instructions.

### Immunofluorescence (IF) Analysis

The IF analysis was performed as previously described.^[^ ^42^ ^]^ Briefly, HCT116 and LS174T cells were seeded on confocal dishes (NEST, #801 001) and incubated overnight. Subsequently, the cells were stimulated with or without 10 µm oxaliplatin for 1 h. After fixation using 4% paraformaldehyde, permeabilization with 0.2% Triton X‐100, and blocking with 5% bovine serum albumin, the cells were incubated with rabbit anti‐RFNG antibody (ThermoFisher Scientific, #PA5‐52844) and mouse anti‐p53 antibody (Santa Cruz Biotechnology, #sc‐126) for 2 h in 4 °C. Followed by incubation with Goat anti‐Rabbit Secondary Antibody (ThermoFisher Scientific, #A‐11008, Alexa Fluor 488) and Goat anti‐Mouse Secondary Antibody (ThermoFisher Scientific, #A‐11005, Alexa Fluor 594) for 1 h. Finally, the cells were stained with 4,6‐diamino‐2‐phenyl indole (DAPI) (ThermoFisher Scientific, #D3571) to visualize the nucleus, and the images were captured using a laser confocal fluorescence microscope (Zeiss, LSM880).

### RNA Extraction and qPCR

Total mRNA extraction was performed using TRIzol reagent (Vazyme, #R401‐01) or the FastPure Cell/Tissue Total RNA Isolation Kit V2 (Vazyme, #RC112) according to the manufacturer's instructions. The extracted mRNA was then reverse transcribed to cDNA using the HiScript III 1st Strand cDNA Synthesis Kit (+gDNA wiper) (Vazyme, #R312). PCR amplification was carried out using Taq Pro Universal SYBR qPCR Master Mix (Vazyme, #Q712‐03). The relative gene expression level was normalized to that of the control gene beta actin (ACTB) using the 2^−∆∆ct^ method. The primers used for qPCR are listed in Table S2 (Supporting Information).

### Immunoblotting and Coimmunoprecipitation (co‐IP)

For immunoblotting, protein samples were collected in RIPA lysis buffer (Thermo Fisher Scientific, #89 901) supplemented with Protease Inhibitor Cocktail (Selleck, #B14002) and Phosphatase Inhibitor Cocktail (Selleck, #B15002). Then, the protein samples were boiled, separated by SDS‒PAGE and transferred to PVDF membranes (Merck Millipore, #IPVH00010). After blocking, the membranes were incubated with targeted primary antibodies overnight at 4 °C. Subsequently, the membranes were incubated with an HRP‐conjugated secondary antibody for 1 h at room temperature. Immunoreactivity was detected using an NcmECL Ultra ECL Chemiluminescence Kit (New Cell & Molecular Biotech, #P10200). For co‐IP, cells were lysed using Pierce IP lysis buffer (Thermo Fisher Scientific, #87 788). The lysates were then incubated with anti‐FLAG M2 Affinity Gel (Sigma Aldrich, #A2220) or the appropriate antibodies along with protein A/G beads at 4 °C overnight. The beads were subsequently washed with IP buffer and used for immunoblot analysis. The antibodies used for immunoblot analysis are listed in Table S3 (Supporting Information).

### Luciferase Assay

Luciferase activity was measured using the TransDetect Double‐Luciferase Reporter Assay Kit (TransGen Biotech, #RF201, Beijing, China) according to the manufacturer's instructions.

### RNA Sequencing Analysis

Total RNA was extracted using TRIzol reagent followed by DNase treatment to eliminate genomic DNA contamination. The mRNA was sequenced using an Illumina HiSeq 2500 sequencing instrument (Shanghai Jingfang Biotechnology Co., Ltd., China). GSEA was performed using GSEA software, and the R programming language was utilized to calculate P values and generate heatmaps.

### ChIP Assay

The ChIP assay was performed according to the manufacturer's protocol (Cell Signaling Technology, #9005). The ChIP PCR primers for CDKN1A were as follows: F, 5′‐ CTTTCACCATTCCCCTACCC −3′; R, 5′‐ AATAGCCACCAGCCTCTTCT −3′. The ChIP PCR primers used for SLC7A11 were as follows: F, 5′‐AGGCTTCTCATGTGGCTGAT‐3′; R, 5′‐TGCATCGTGCTCTCAATTCT‐3′.

### Patient Samples

The study was approved by the Ethics Committee of The First Affiliated Hospital, Sun Yat‐sen University, and was conducted in accordance with the ethical guidelines of the Declaration of Helsinki. QPCR and immunoblotting analyses were conducted to assess RFNG expression in tumor tissues and paired adjacent normal tissues from 32 patients with CRC. Additionally, immunohistochemistry (IHC) was performed to evaluate RFNG levels in tumor tissues and paired adjacent normal tissues from 77 patients with CRC. Sixty‐two CRC patient samples from p53 WT patients receiving oxaliplatin‐based chemotherapy were collected to explore the clinical relevance of RFNG pS255‐regulated p53 phosphorylation and CDKN1A and SLC7A11 protein levels. For organoid treatment, human CRC organoids were isolated, generated, and cultured human colorectal cancer organoids were performed as described previously.^[^ ^43^ ^]^ ShNT or shRFNG lentivirus was used to infect organoids, which were then treated with or without 10 µm oxaliplatin for the indicated times. Relative proliferation was analyzed using Cell Titer‐Glo‐Assay (Promega, #G9681) according to the manufacturer's protocol. All tissue samples were obtained from The First Affiliated Hospital, Sun Yat‐sen University, with the consent of the patients.

### Animal Experiments

The experimental procedures involving animals were approved by the Institutional Animal Care and Use Committee of the Sun Yat‐sen University. All animal experiments were conducted in accordance with the National Institute of Health Guide for the Care and Use of Laboratory Animals, and mice were maintained under specific pathogen‐free (SPF) conditions. All the mice were purchased from Guangdong Gem Pharmatech (Guangdong, China). For the BALB/c nude mouse subcutaneous xenograft model, six‐week‐old female BALB/c nude mice were randomly assigned to different groups. The cells were subcutaneously injected into the dorsal region of mice. Once the tumors approximately reached a volume of 100 mm^3^, randomization was performed by dividing the tumor‐bearing mice with similar tumor burdens into the control and drug treatment groups. The control group received vehicle treatment (*n* = 5, i.p., every three days), whereas the treatment group received oxaliplatin (*n* = 5, i.p., 7.5 mg kg^−1^, every three days). Tumor volume was measured every four days, and tumors were excised upon reaching a volume of 1000 mm^3^ or at the end of the study. For the patient‐derived xenograft (PDX) model, fresh tumor fragments obtained from patients with CRC were subcutaneously implanted into NOD‐SCID mice during surgery. The tumors were washed with PBS and transferred to DMEM/F12 basic medium. Whole tumors were minced into small patches that could pass through a needle bore and then injected subcutaneously into mice. Randomization was performed by dividing tumor‐bearing mice with similar tumor burdens into control and experimental groups for oxaliplatin treatment. Tumors were excised when they reached a volume of 1000 mm^3^ or at the end of the study.

### IHC Staining

IHC staining was performed as previously described.^[^ ^44^, ^45^ ^]^ Briefly, tissue slices were deparaffinized by immersion in xylene twice for 10 min each, followed by hydration in an alcohol gradient. Antigen retrieval was performed by placing the tissues in sodium citrate repair solution (Beyotime, #P0083). The slides were incubated with the desired antibodies overnight at 4 °C. The following day, rapid color development was achieved using DAB, and expression levels were estimated using an IHC score. The antibodies used for IHC were RFNG (Thermo Fisher Scientific, #PA5‐52844), p53 pS15 (Cell Signaling Technology, #9284), CDKN1A (Cell Signaling Technology, #2947), and SLC7A11 (HUABIO, #HA600098).

### Statistical Analysis

Statistical analysis and graphing were performed using the GraphPad Prism 8 software. Student's t‐test was used for comparisons between two independent groups, while one‐way ANOVA or two‐way ANOVA with Bonferroni correction was used for comparisons among more than two groups. The log‐rank test was used to compare the patient survival rates. *P* < 0.05 was considered to indicate statistical significance.

## Conflict of Interest

The authors declare no conflict of interest.

## Author Contributions

Z.W. and W.H. conceived and designed the project. M.S. contributed to the extensive discussions. Y.D. performed most of the experiments. X.Z. performed some key experiments. X.W., J.Q., L.Y., and Y.W. performed parts of involved experiments. Y.D. and Z.W. wrote the manuscript. Z.W. supervised the project. The final draft of the study was approved by all authors.

## Supporting information

Supporting Information

## Data Availability

The data that support the findings of this study are available from the corresponding author upon reasonable request.
