# Supplementary material for: MAPK Signaling‐Mediated RFNG Phosphorylation and Nuclear Translocation Restrain Oxaliplatin‐Induced Apoptosis and Ferroptosis
Source: Adv Sci (Weinh). 2024 Aug 9;11(38):2402795. doi: 10.1002/advs.202402795 (PMC11481204; doi:10.1002/advs.202402795)
Supplement: Supplementary file 1 — Supporting Information [file ADVS-11-2402795-s001.docx]

Supplementary Information

**MAPK Signaling-Mediated RFNG Phosphorylation and Nuclear Translocation Restrain Oxaliplatin-Induced Apoptosis and Ferroptosis**

*Yuqin Di, Xiang Zhang, Xiangqiong Wen, Jiale Qin, Lvlan Ye, Youpeng Wang, Mei Song^*^, Ziyang Wang^*^, Weiling He^*^*

This supporting information includes:

1) Seven Supplementary Figures

2) Three Supplementary Tables

**Supplementary Figures**

**
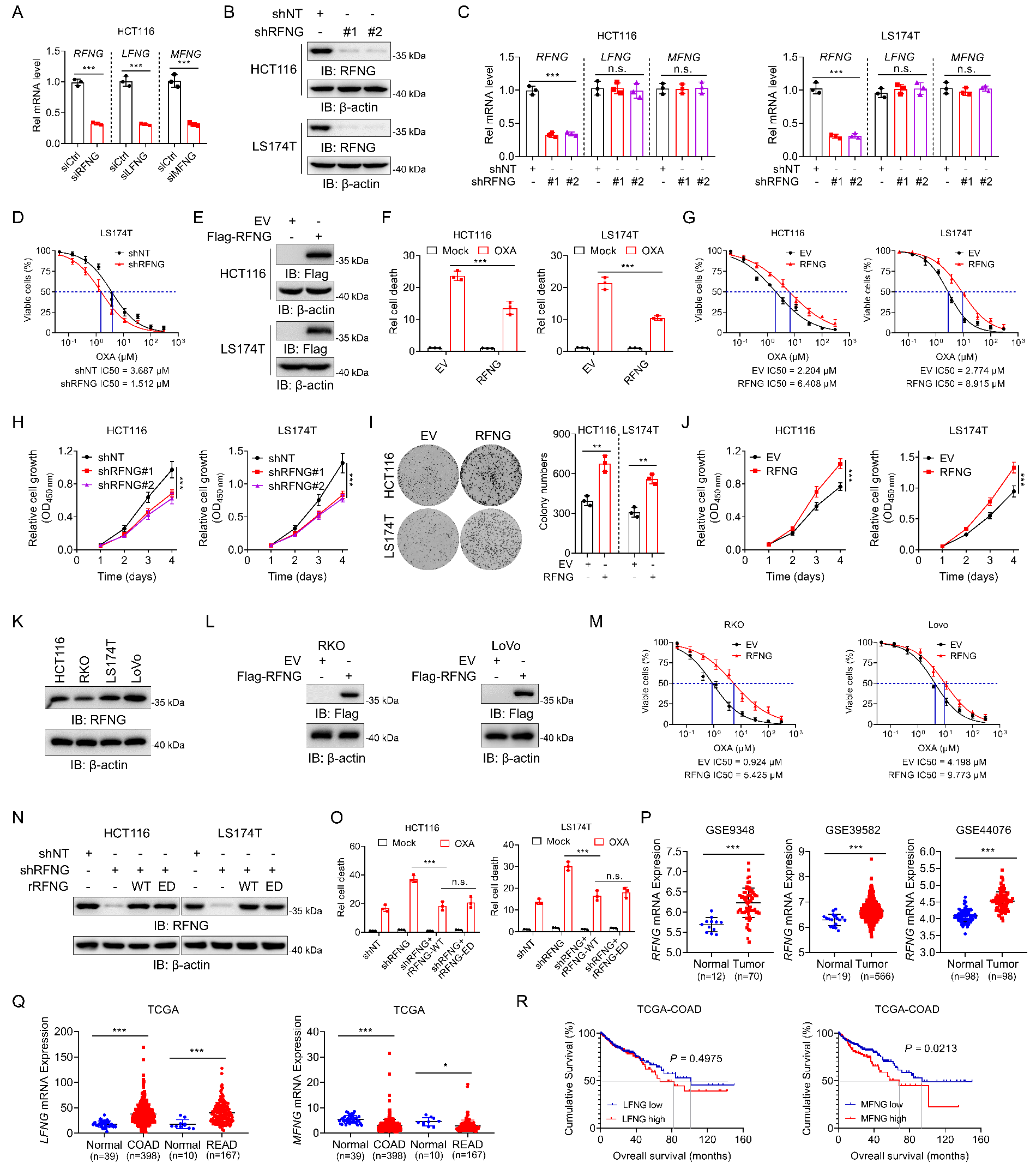
**

**Figure S1. RFNG promotes oxaliplatin chemoresistance and is a prognostic biomarker in CRC.** (A) QPCR analysis of the expression of *RFNG*, *LFNG*, or *MFNG* in the indicated cells. (B) Immunoblotting analysis to assess the knockdown efficiency for shRFNG in HCT116 and LS174T cells. (C) QPCR analysis of the expression of *RFNG*, *LFNG*, or *MFNG* in HCT116 and LS174T cells stably expressing shNT, shRFNG-1, or shRFNG-2. (D) LS174T cells stably expressing shNT, shRFNG-1 or shRFNG-2 were treated with or without 20 μM OXA for 48 hours, and the IC50 was assessed. (E) Immunoblotting analysis confirming the efficiency of RFNG overexpression in HCT116 and LS174T cells. (F, G) HCT116 and LS174T cells stably expressing EV or RFNG were treated with or without 20 μM OXA. Cell death was measured after treatment for 24 hours (F), and the IC50 was assessed after treatment for 48 hours (G). (H) Cell proliferation curves were generated to assess the proliferation of HCT116 and LS174T cells stably expressing EV or RFNG. (I, J) Colony formation assays (I) and cell proliferation curves (J) were conducted to assess the proliferation of HCT116 and LS174T cells stably expressing EV or RFNG. (K) Immunoblotting analysis of RFNG expression in HCT116, RKO, LS174T, and LoVo cells. (L) Immunoblotting analysis confirming the efficiency of RFNG overexpression in RKO and LoVo cells. (M) RKO and LoVo cells stably expressing EV or RFNG were treated with OXA, and the IC50 was assessed after treatment for 48 hours. (N) Immunoblotting analysis of RFNG expression in shNT, shRFNG, or shRFNG cells rescued with rRFNG-WT or rRFNG-ED in HCT116 and LS174T cells. (O) HCT116 and LS174T cells stably expressing shNT, shRFNG or shRFNG rescued with rRFNG-WT or rRFNG-ED were treated with or without 20 μM OXA for 24 hours, and cell death was assessed. (P) The mRNA expression of *RFNG* in normal and tumor samples from the GEO datasets GSE9348, GSE39582, and GSE44076. (Q) The mRNA expression of *LFNG* and *MFNG* in normal and tumor samples from the TCGA COAD and READ databases. (R) Kaplan‒Meier analysis of overall survival according to LFNG (left) or MFNG (right) expression in CRC patient samples from the TCGA COAD database. **P* <0.05, ***P* <0.01, ****P* < 0.001, n.s. = non-significant (two-tailed t test (A, F, I, P, Q), two-way ANOVA (H, J), one-way ANOVA (C, O), or log-rank test (R)).

**
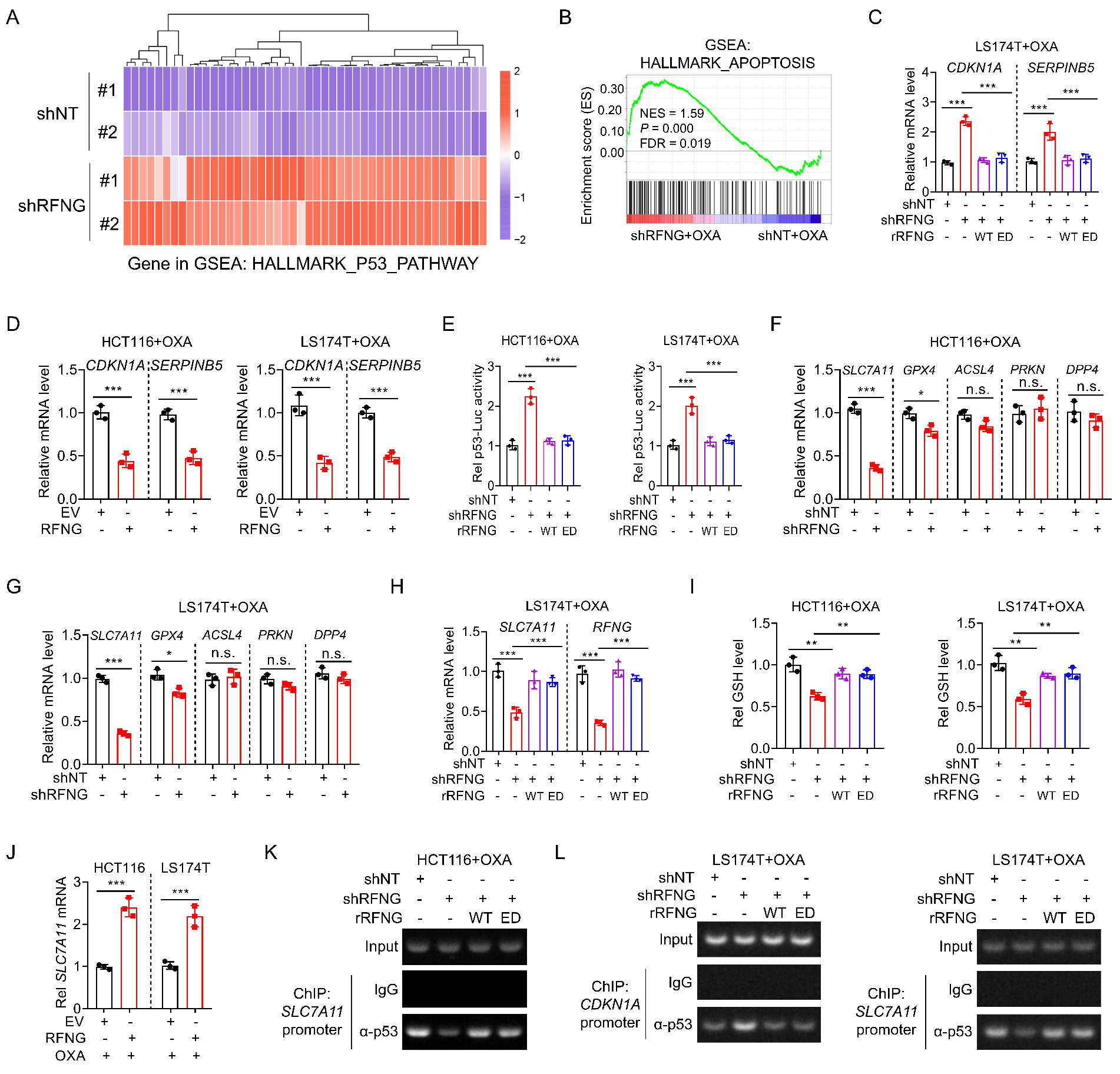
**

**Figure S2. RFNG regulates the expression of CDKN1A and SLC7A11.** (A) Heatmap displaying the expression of RFNG knockdown-regulated p53 pathway genes according to GSEA. (B) GSEA showed that the apoptosis pathway was enriched among the upregulated pathways in shRFNG cells. (C) LS174T cells stably expressing shNT, shRFNG, or shRFNG were rescued with rRFNG-WT or rRFNG-ED, and qPCR analysis of the mRNA expression of *CDKN1A* and *SERPINB5* after treatment with 20 μM OXA for 12 hours was performed. (D) HCT116 and LS174T cells stably expressing EV and RFNG, and qPCR analysis of the mRNA expression of *CDKN1A* and *SERPINB5* was performed after treatment with 20 μM OXA for 12 hours. (E) p53-luc activity was performed in HCT116 and LS174T cells stably expressing shNT, shRFNG, or shRFNG rescued with rRFNG-WT or rRFNG-ED and treated with 20 μM OXA for 12 hours. (F, G) HCT116 (F) and LS174T (G) cells stably expressing shNT and shRFNG, and qPCR analysis of the mRNA expression of indicated genes after treatment with 20 μM OXA for 12 hours was performed. (H) LS174T cells stably expressing shNT, shRFNG, or shRFNG were rescued with rRFNG-WT or rRFNG-ED, and qPCR analysis of *SLC7A11* mRNA expression after treatment with 20 μM OXA for 12 hours was performed. (I) HCT116 and LS174T cells stably expressing shNT, shRFNG, or shRFNG were rescued with rRFNG-WT or rRFNG-ED, and GSH levels were assessed after treatment with 20 μM OXA for 12 hours. (J) HCT116 and LS174T cells stably expressing EV and RFNG were subjected to qPCR analysis of *SLC7A11* mRNA expression after treatment with 20 μM OXA for 12 hours. (K) HCT116 cells stably expressing shNT, shRFNG, or shRFNG were rescued with rRFNG-WT or rRFNG-ED, and ChIP PCR analysis of p53 binding at the *SLC7A11* promoters after treatment with 20 μM OXA for 12 hours was performed. (L) LS174T cells stably expressing shNT, shRFNG, or shRFNG were rescued with rRFNG-WT or rRFNG-ED, and ChIP PCR analysis of p53 binding at the *CDKN1A* and *SLC7A11* promoters after treatment with 20 μM OXA for 12 hours was performed. ***P* <0.01, ****P* < 0.001 (one-way ANOVA (C, E, H, I), or two-tailed t test (D, F, G, J)).

**
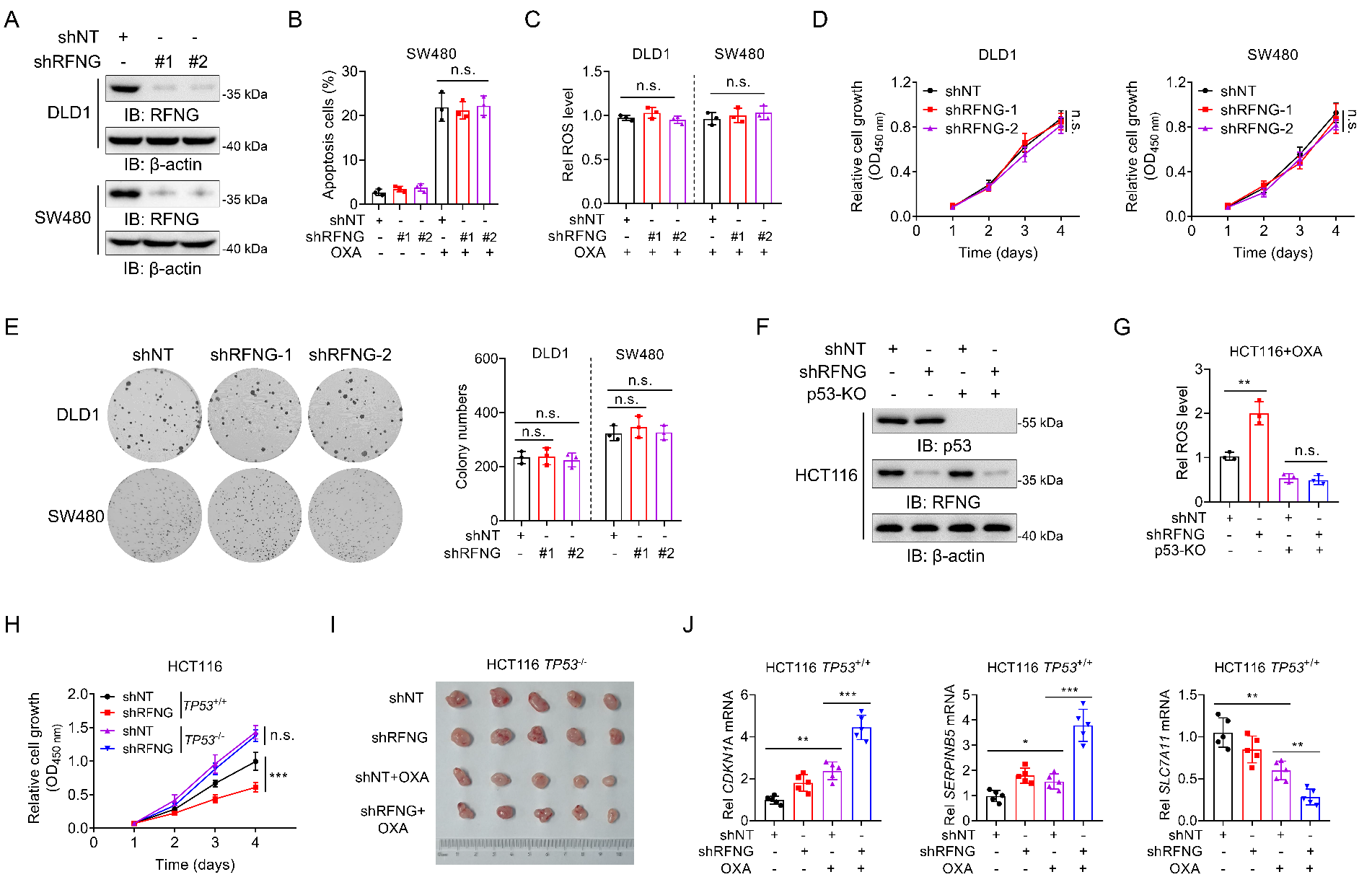
**

**Figure S3. The promotion of chemoresistance by RFNG is dependent on the presence of wild-type p53.** (A) Immunoblotting analysis to assess the knockdown efficiency of shRFNG in DLD1 and SW480 cells. (B, C) DLD1 and SW480 cells stably expressing shNT, shRFNG-1, or shRFNG-2 were treated with or without 50 μM OXA. The number of apoptotic cells after treatment for 24 hours (B) and the ROS levels after treatment for 12 hours (C) were assessed. (D, E) Cell proliferation curves (D) and colony formation assays (E) were conducted to assess the proliferation of DLD1 and SW480 cells stably expressing shNT, shRFNG-1, or shRFNG-2. (F) Immunoblotting analysis of the expression of the indicated proteins in *TP53*^+/+^ and *TP53*^-/-^ HCT116 cells stably expressing shNT or shRFNG. (G) HCT116 *TP53*^+/+^ or *TP53*^-/-^ cells were treated with 20 μM OXA, and the ROS levels were assessed. (H) Cell proliferation curves were generated to assess the proliferation of *TP53*^+/+^ and *TP53*^-/-^ HCT116 cells stably expressing shNT or shRFNG. (I) HCT116 *TP53*^-/-^ cells stably expressing shNT or shRFNG were subcutaneously injected into nude mice, followed by i.p. injection of OXA (7.5 mg/kg) or vehicle (n = 5). Images of the tumors on the termination day are shown. (J) QPCR analysis of the mRNA expression of *CDKN1A*, *SERPINB5*, and *SLC7A11* in the indicated xenograft tumors. **P* <0.05, ***P* <0.01, ****P* < 0.001, n.s. = nonsignificant (one-way ANOVA (B, C, E, G, J), or two-way ANOVA (D, H)).

**
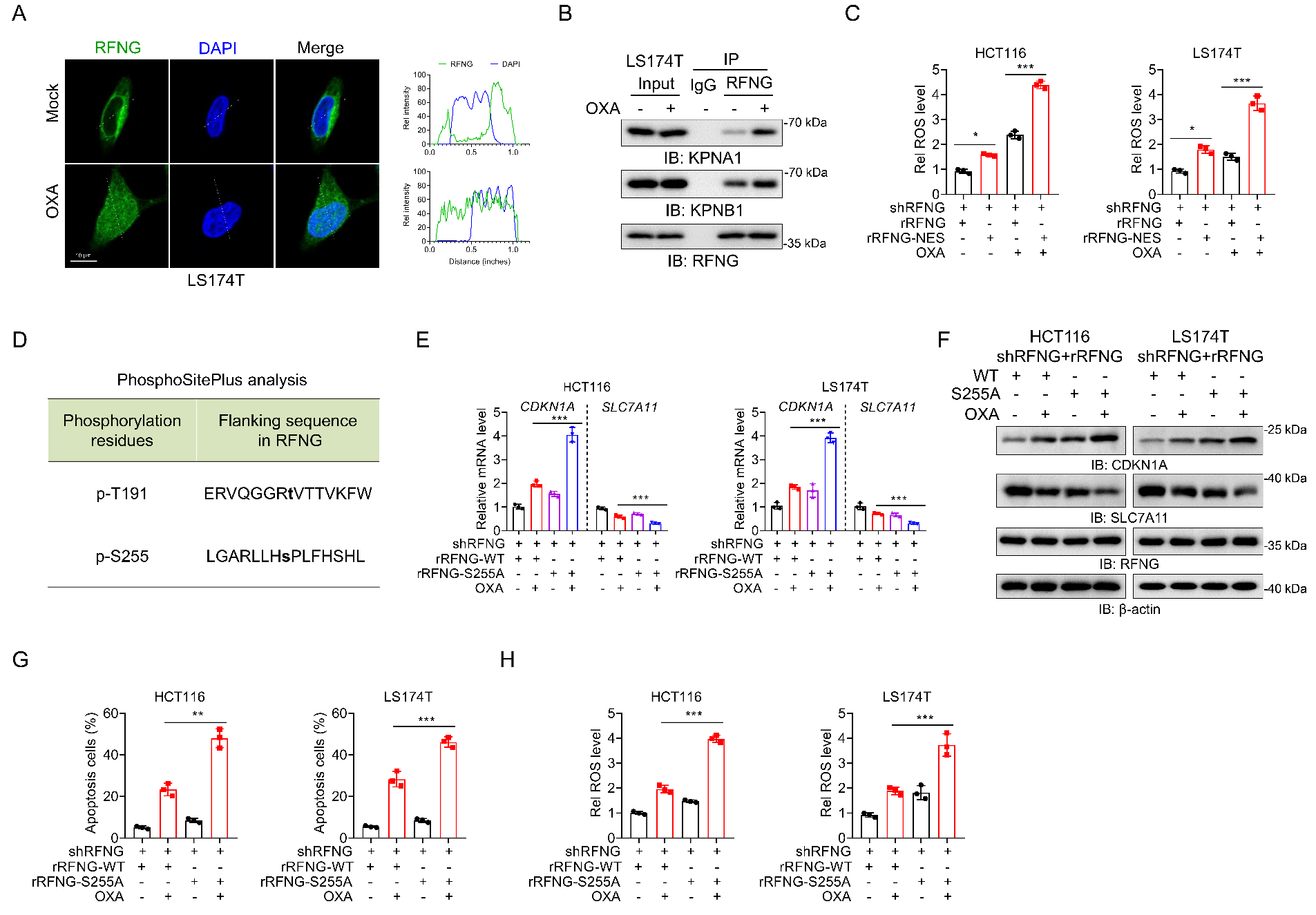
**

**Figure S4. Oxaliplatin treatment induces RFNG S255 phosphorylation and nuclear translocation.** (A) LS174T cells were treated with or without 10 μM OXA for 1 hour, and then immunofluorescence (IF) staining was performed. RFNG localization was indicated by an anti-RFNG antibody, and nuclei were labeled with DAPI (left). Signal intensities and distances were quantified (right). Scale bar, 10 μm. (B) LS174T cells were treated with or without 10 μM OXA for 1 hour, after which co-IP was performed. (C) RFNG-knockdown HCT116 and LS174T cells stably expressing rRFNG-WT or rRFNG-NES were treated with or without 20 μM OXA, and relative ROS levels were assessed. (D) The phosphorylated residues of RFNG (T191, S255) were identified using the PhosphoSitePlus database (<https://www.phosphosite.org/homeAction>). (E, F) RFNG-knockdown HCT116 and LS174T cells stably expressing rRFNG-WT or rRFNG-S255A were treated with or without 10 μM OXA for 12 hours, and qPCR analysis of the mRNA expression (E) and immunoblotting analysis the protein expression (F) of CDKN1A and SLC7A11 were performed. (G, H) RFNG-knockdown HCT116 and LS174T cells stably expressing rRFNG-WT or rRFNG-S255A were treated with or without 20 μM OXA, and the number of apoptotic cells (G) and relative ROS levels (H) were assessed. **P* <0.05, ***P* <0.01, ****P* < 0.001 (one-way ANOVA (C, E, G, H)).

**
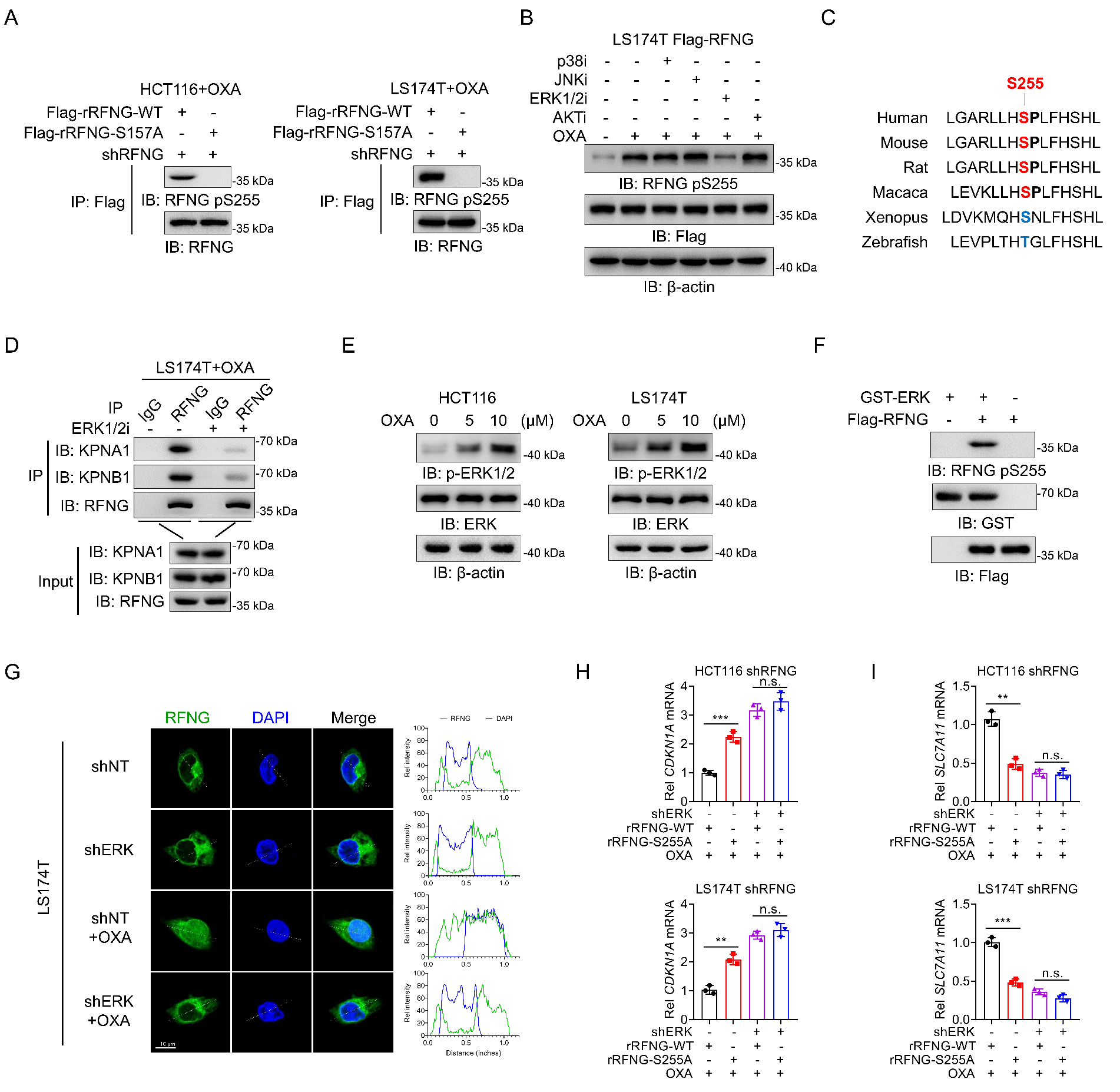
**

**Figure S5. ERK phosphorylates RFNG to promote its translocation.** (A) RFNG-knockdown HCT116 and LS174T cells stably expressing rRFNG-WT or rRFNG-S255A were treated with 10 μM OXA for 1 hour, and co-IP was performed. (B) LS174T cells stably expressing Flag-RFNG were treated with or without the indicated inhibitors for 12 hours, followed by treatment with 10 μM OXA for 1 hour. Immunoblotting analysis was subsequently performed. (C) Sequence alignment of the RFNG-phosphorylated S255 residue in the indicated species. (D) LS174T cells were treated with or without ERK1/2i for 12 hours, followed by 10 μM OXA for 1 hour. Co-IP was subsequently performed. (E) HCT116 and LS174T cells were treated with 5 μM or 10 μM OXA for 1 hour, and cells were collected for immunoblotting analysis. (F) An in vitro kinase assay was performed by mixing GST-ERK and Flag-RFNG. (G) LS174T cells stably expressing shNT and shERK were treated with or without 10 μM OXA for 1 hour. IF were performed. Scale bar, 10 μm. (H, I) HCT116 and LS174T cells stably expressing shNT or shERK were infected with rRFNG-WT or rRFNG-S255A and treated with 20 μM OXA for 12 hours. QPCR analysis of the mRNA expression of *CDKN1A* (H) and *SLC7A11* (I). ***P* < 0.01, ****P* < 0.001, n.s. = nonsignificant (one-way ANOVA (H, I)).

**
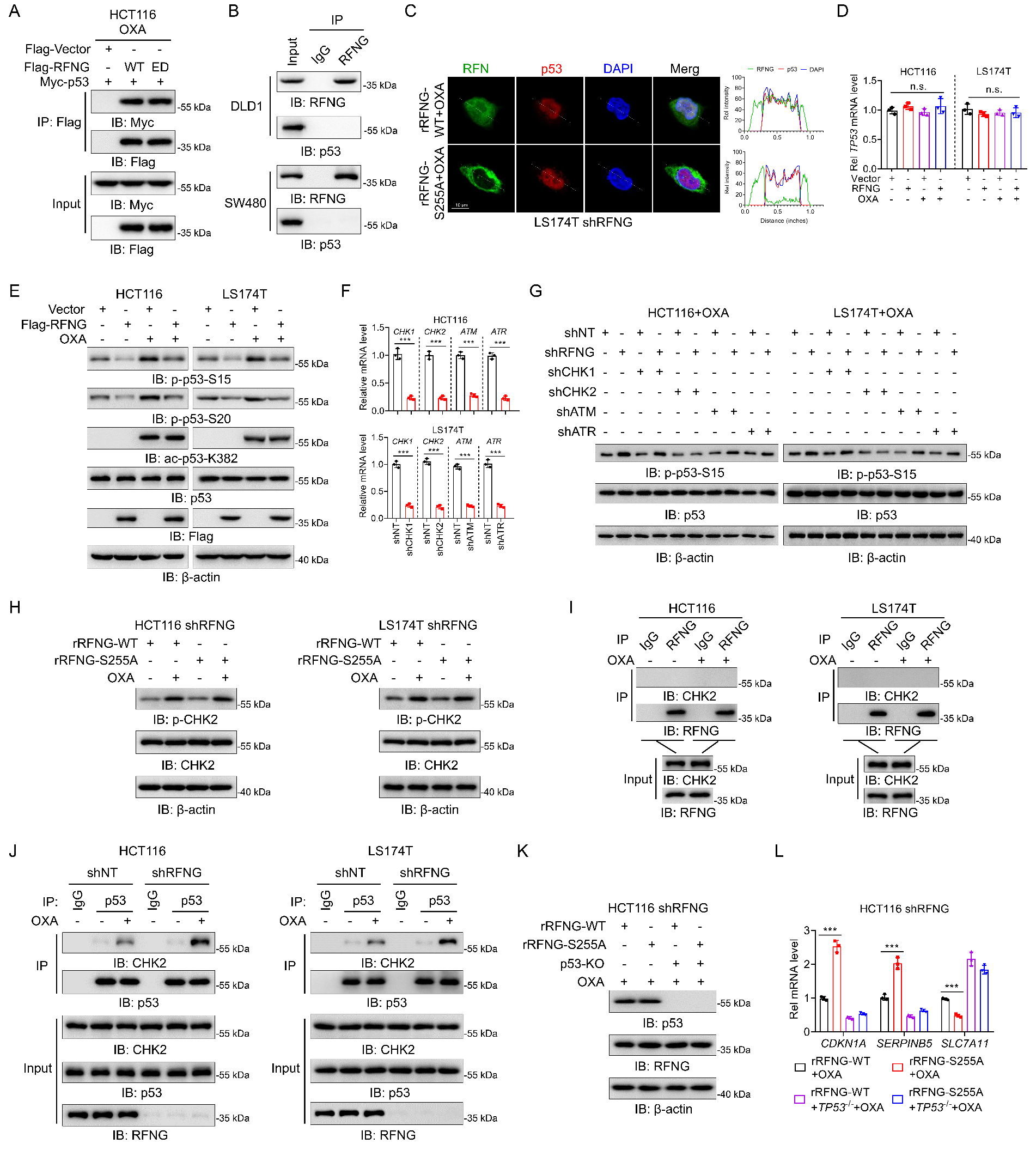
**

**Figure S6. S255-phosphorylated RFNG (pS255) binds to p53 and inhibits its activity.** (A) HCT116 and LS174T cells stably expressing Flag-vector, RFNG-WT or RFNG-ED were transfected with Myc-p53 and then treated with or without 10 μM OXA for 1 hour. Co-IP was subsequently performed. (B) RFNG was immunoprecipitated with indicated antibodies in DLD1 and SW480 cells, immunoblotting analysis was subsequently performed. (C) RFNG-knockdown LS174T cells stably expressing rRFNG-WT or rRFNG-S255A were treated with 10 μM OXA for 1 hour. IF analysis was performed. Scale bar, 10 μm. (D) HCT116 and LS174T cells stably expressing vector and RFNG were treated with or without 10 μM OXA for 12 hours, and qPCR analysis of the mRNA expression of *TP53* was performed. (E) HCT116 and LS174T cells stably expressing vector and RFNG were treated with or without 10 μM OXA for 1 hour, after which immunoblotting analysis was performed. (F) QPCR analysis to assess the knockdown efficiency for indicated shRNAs in HCT116 and LS174T cells. (G) HCT116 and LS174T cells stably expressing shNT and shRFNG were infected with lentivirus expressing shNT or indicated shRNAs, and then cells were treated with or without 10 μM OXA for 1 hour, after which immunoblotting analysis was performed. (H) RFNG-knockdown HCT116 and LS174T cells stably expressing rRFNG-WT or rRFNG-S255A were treated with or without 10 μM OXA for 1 hour, and immunoblotting analysis was performed. (I) HCT116 and LS174T cells were treated with or without 10 μM OXA for 1 hour, and co-IP was performed. (J) HCT116 and LS174T cells stably expressing shNT or shRFNG were treated with or without 10 μM OXA for 1 hour. Co-IP was subsequently performed. (K) RFNG-depleted HCT116 *TP53*^+/+^ or *TP53*^-/-^ cells were infected with rRFNG-WT or rRFNG-S255A, and immunoblotting analysis was performed. (L) RFNG-depleted HCT116 *TP53*^+/+^ or *TP53*^-/-^ cells were infected with rRFNG-WT or rRFNG-S255A and then treated with 20 μM OXA for 12 hours. QPCR analysis of the mRNA expression of *CDKN1A*, *SERPINB5* and *SLC7A11*. ****P* < 0.001, n.s. = nonsignificant (one-way ANOVA (D, L), or two-tailed t test (F)).

**
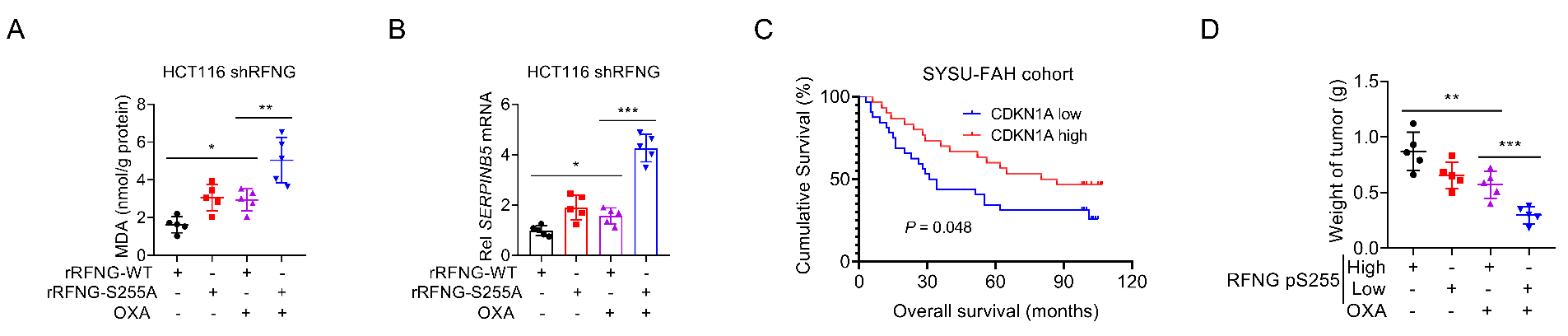
**

**Figure S7. The level of phosphorylated RFNG was negatively correlated with the efficacy of chemotherapy.** (A) MDA levels were assessed in xenograft tumors. (B) QPCR analysis of the mRNA expression of *SERPINB5* in the indicated xenograft tumors. (C) Kaplan‒Meier analysis of overall survival according to CDKN1A protein levels in human CRC samples. (D) Oxaliplatin (7.5 mg/kg) treatment was tested in PDX models, and tumor weights are shown. **P* <0.05, ****P* < 0.001 (one-way ANOVA (A, B, D) or log-rank test (C)).

**Supplementary Tables**

**Table S1. The sequences of siRNAs, shRNAs and sgRNAs.**

| Identifier | Sequences (5'-3') |
| --- | --- |
| siRFNG | CCGUGGAGUAUGACAAGUUCAUUGA |
| siLFNG | GGGUCACUUCAUGAAUACGGCUGAG |
| siMFNG | CAGCUACACGAUGUCUUCAUUGCAG |
| shRFNG-1 | GTCAAGTTCTGGTTTGCTACT |
| shRFNG-2 | GTCTATCCATTGTCTTCTGTA |
| shERK | CAAAGTTCGAGTAGCTATCAA |
| sgp53-1 | CCCCGGACGATATTGAACAA |
| sgp53-2 | CCGGTTCATGCCGCCCATGC |
| shCHK1 | GTGGTTTATCTGCATGGTATT |
| shCHK2 | CGCCGTCCTTTGAATAACAAT |
| shATM | CTCTTACAACACCACCGAGAT |
| shATR | GCGATACTGTTGAATGTGAAT |

**Table S2. Quantitative real-time qPCR primers**

| Gene name | Forward (5'-3') | Reverse (5'-3') |
| --- | --- | --- |
| *ACTB* | GCGTGACATTAAGGAGAAG | GAAGGAAGGCTGGAAGAG |
| *RFNG* | CGCCAGCAGACGTTTATCTTC | CCGAGCAGTTGGTGTTGATG |
| *LFNG* | GTCAGCGAGAACAAGGTGC | GATCCGCTCAGCCGTATTCAT |
| *MFNG* | ACGAGGCTGGTACAGTTCTG | GATGTGTCCATGAAACGGGAG |
| *CDKN1A* | TGTCCGTCAGAACCCATGC | AAAGTCGAAGTTCCATCGCTC |
| *SERPINB5* | AATTCGGCTTTTGCCGTTGAT | TGTCACCTTTAGCACCCACTT |
| *SLC7A11* | TCTCCAAAGGAGGTTACCTGC | AGACTCCCCTCAGTAAAGTGAC |
| *GPX4* | GAGGCAAGACCGAAGTAAACTAC | CCGAACTGGTTACACGGGAA |
| *ACSL4* | CATCCCTGGAGCAGATACTCT | TCACTTAGGATTTCCCTGGTCC |
| *PRKN* | GTGTTTGTCAGGTTCAACTCCA | GAAAATCACACGCAACTGGTC |
| *DPP4* | TACAAAAGTGACATGCCTCAGTT | TGTGTAGAGTATAGAGGGGCAGA |
| *CHK1* | ATATGAAGCGTGCCGTAGACT | TGCCTATGTCTGGCTCTATTCTG |
| *CHK2* | TTATCTGCCTTAGTGGGTATCCA | CTGTCGTAAAACGTGCCTTTG |
| *ATM* | TTGATCTTGTGCCTTGGCTAC | TATGGTGTACGTTCCCCATGT |
| *ATR* | TCCCTTGAATACAGTGGCCTA | TCCTTGAAAGTACGGCAGTTC |

**Table S3. Antibodies used for IB and IP analyses**

| Antibody | Source | Company |
| --- | --- | --- |
| RFNG | Rabbit pAb (PA5-52844) | ThermoFisher Scientific |
| β-actin | Mouse mAb (A1978) | Sigma Aldrich |
| Flag-tag | Mouse mAb (F1804) | Sigma Aldrich |
| CDKN1A | Rabbit mAb (2947) | Cell Signaling Technology |
| SERPINB5 | Rabbit pAb (A1179) | ABclonal Technology |
| Cle-Caspase-3 | Rabbit mAb (9664) | Cell Signaling Technology |
| Cle-Caspase-7 | Rabbit mAb (42542) | Cell Signaling Technology |
| SLC7A11 | Rabbit mAb (12691) | Cell Signaling Technology |
| p53 | Mouse mAb (sc-126) | Santa Cruz Biotechnology |
| Tubulin | Mouse mAb (sc-166729) | Santa Cruz Biotechnology |
| Histone H3 | Rabbit mAb (9715) | Cell Signaling Technology |
| KPNA1 | Rabbit pAb (A1179) | ABclonal Technology |
| KPNB1 | Rabbit mAb (A23235) | ABclonal Technology |
| phospho-Ser/Thr | Rabbit pAb (ab117253) | Abcam |
| phospho-Tyr | Mouse mAb (sc-508) | Santa Cruz Biotechnology |
| pan-Acetylation | Mouse mAb (66289-1-Ig) | Proteintech Group |
| RFNG pS255 | Rabbit pAb | HUABIO |
| ERK | Rabbit mAb (9108) | Cell Signaling Technology |
| p-ERK1/2 | Rabbit mAb (4370) | Cell Signaling Technology |
| Myc-tag | Rabbit mAb (2278) | Cell Signaling Technology |
| HA-tag | Rabbit mAb (3724) | Cell Signaling Technology |
| p-p53 (S15) | Rabbit mAb (9284) | Cell Signaling Technology |
| p-p53 (S20) | Rabbit mAb (9287) | Cell Signaling Technology |
| ac-p53 (K382) | Rabbit mAb (2525) | Cell Signaling Technology |
| CHK2 | Rabbit mAb (A19543) | ABclonal Technology |
